# Temporal trajectories of artificial radiocaesium ^137^Cs in French rivers over the nuclear era reconstructed from sediment cores

**DOI:** 10.1038/s41598-024-64505-7

**Published:** 2024-06-20

**Authors:** Frédérique Eyrolle, Pierre-Alexis Chaboche, Hugo Lepage, Valérie Nicoulaud Gouin, Patrick Boyer, Anne De Vismes, Gabrielle Seignemartin, Dominique Badariotti, François Chabaux, Maxime Chastanet, David Claval, Yoann Copard, Alexandra Coynel, Maxime Debret, Claire Delus, Cassandra Euzen, Thomas Gardes, Franck Giner, Rodolfo Gurriaran, Christian Grenz, Cécile Grosbois, Laurence Lestel, Benoît Losson, Laurence Mansuy-Huault, Emmanuelle Montarges-Pelletier, Amandine Morereau, Brice Mourier, David Mourier, Vincent Ollive, Laure Papillon, Jorg Schafer, Laurent Schmitt, Richard Sempere, Thierry Winiarski, Mathilde Zebracki, Olivier Evrard

**Affiliations:** 1 PSE-ENV, STAAR/LRTA, Institut de Radioprotection et de Sûreté Nucléaire, BP 3, 13115 Saint Paul Lez Durance, France; 2 International Research Fellow of Japan Society for the Promotion of Science (Postdoctoral Fellowships for Research in Japan (Standard)), Tokyo, Japan; 3 Institute of Environmental Radioactivity, Fukushima University, Fukushima, Japan; 4 PSE-ENV, SAME/LMRE, Institut de Radioprotection et de Sûreté Nucléaire, Bois des Rames, 91400 Orsay, France; 5 CNRS, ENTPE, UMR5023 LEHNA, Univ Lyon, Université Claude Bernard Lyon 1, 69518 Vaulx-en-Velin, France; 6 Laboratoire Image Ville Environnement (LIVE UMR 7362), CNRS, ENGEES, Université de Strasbourg, Strasbourg, France; 7 Institut Terre et Environnement de Strasbourg, CNRS, Université de Strasbourg, 5 rue René Descartes, 67000 Strasbourg, France; 8 EPOC-TGM, UMR CNRS 5805, Pessac, France; 9 CNRS-M2C Lab. Department Geosciences and Environment, University of Rouen-Normandy, 76821 Mont Saint Aignan, France; 10 EA 7304 “LOTERR”, Université de Lorraine, Nancy, France; 11 CNRS, LCE, UMR 7376, Aix-Marseille Université, Marseille, France; 12 UR 6293 Géohydrosystèmes Continentaux (GéHCO), Université de Tours, Parc de Grandmont, Cedex, 37200 Tours, France; 13 METIS - Milieux Environnementaux, Transferts et Interactions dans les Hydrosystèmes et les Sols, UMR 7619, Sorbonne Université, 75252 Paris, France; 14 Université de Lorraine, CNRS, LIEC, 54000 Nancy, France; 15 ZAM - Zone Atelier du Bassin de La Moselle [LTSER France], Nancy, France; 16 CNRS, LCE, UMR 7376, Ocean Sciences Institute, Aix-Marseille Université, Aix-en-Provence, France; 17 Institut de Radioprotection et de Sûreté Nucléaire, PSE-ENV/SPDR/LT2S, 13115 Saint-Paul-Lez-Durance, France; 18 Laboratoire des Sciences du Climat et de l’Environnement (LSCE-IPSL), UMR 8212 (CEA/CNRS/UVSQ), Université Paris-Saclay, CEA Saclay – l’Orme des Merisiers, 91191 Gif-Sur-Yvette, France

**Keywords:** Sediment cores, Rivers, Radiocaesium, Radioactivity, Trajectories, Resiliency, Biogeochemistry, Environmental sciences, Hydrology

## Abstract

^137^Cs is a long-lived man-made radionuclide introduced in the environment worldwide at the early beginning of the nuclear Era during atmospheric nuclear testing’s followed by the civil use of nuclear energy. Atmospheric fallout deposition of this major artificial radionuclide was reconstructed at the scale of French large river basins since 1945, and trajectories in French nuclearized rivers were established using sediment coring. Our results show that ^137^Cs contents in sediments of the studied rivers display a large spatial and temporal variability in response to the various anthropogenic pressures exerted on their catchment. The Loire, Rhone, and Rhine rivers were the most affected by atmospheric fallout from the global deposition from nuclear tests. Rhine and Rhone also received significant fallout from the Chernobyl accident in 1986 and recorded significant ^137^Cs concentrations in their sediments over the 1970–1985 period due to the regulatory releases from the nuclear industries. The Meuse River was notably impacted in the early 1970s by industrial releases. In contrast, the Seine River display the lowest ^137^Cs concentrations regardless of the period. All the rivers responded similarly over time to atmospheric fallout on their catchment, underlying a rather homogeneous resilience capacity of these river systems to this source of contamination.

## Introduction

Based on the location of the testing sites and the yield of detonations, it is estimated that the Northern Hemisphere received 75% of the total radioactive atmospheric fallout even though uncomplete data sets may be available for the southern Hemisphere for the period post-1963^1^. According to Ref.^2^, the major global atmospheric circulation has concentrated the deposits in temperate regions, particularly in the band between 40 and 50 degrees of latitude, where Western Europe is located. The last atmospheric explosion, with a power of 0.6 Mt, took place in Lop Nor test site (China) on October 16, 1980^2–6^. Numerous artificial radionuclides were produced during the period of nuclear tests, among them activation products such as tritium and radiocarbon and long-lived fission products including plutonium isotopes (T_1/2_ from 14y for ^241^Pu to several 10^4^y for ^239^Pu), ^90^Sr (T_1/2_ = 28.5y) and ^137^Cs (T_1/2_ = 30.1y). Initially introduced in the environment during atmospheric nuclear testing from 1945 to 1980, radiocaesium (^137^Cs) was distributed at the global scale in both hemispheres and is consequently one of the most studied artificial radionuclides found in various environmental compartments all around the world since the early beginning of the nuclear era.

A peak in radioactive fallout onto the Earth surface was registered in 1963 and led to a long-term environmental imprint with the circulation of radionuclides between environmental compartments, including riverine sediments. These geological imprints of human activities would define the onset of the Anthropocene era. Within the geoscientist community, the ^137^Cs emission peak from nuclear tests was widely used, combined to the naturally occurring radionuclide ^210^Pb_xs_, to date sediment cores from the last one hundred years^7^, mainly because of its affinity for soil and sediment particles making it a valuable sediment tracer^8–17^. ^137^Cs as most of trace elements easily adsorb onto naturally occurring solid particles, and its mass concentration most generally increases when the grain size of particles decreases mainly due to the associated enhancement of the reactive surface aera and due to the mineralogical nature of fine fraction (clay minerals)^18^.

Additional ^137^Cs was introduced into the environment at a regional scale due to nuclear accidents. ^137^Cs fallout from the Chernobyl accident (USSR) which occurred on 26^th^ April 1986 was detected over a large part of Europe^19–22^. A large heterogeneity of such atmospheric deposits was observed in relation to both air masses circulation and rainfall occurrence over Europe during the days that followed the accident. In France, a longitudinal attenuation of ^137^Cs contents in soils from East to West was most generally observed, even though enriched ^137^Cs hotspots were directly correlated to rainfall intensity or snow covering in mountainous areas^23–26^. The Fukushima nuclear accident (11 March 2011, Japan) is the second nuclear power plant disaster in history classified at level 7, the highest on the International Nuclear Event Scale (INES). It presents the same level of severity as the Chernobyl disaster, in particular following the large volume of radioactive releases that took place into the Pacific Ocean and on soils of Northeastern Japan^27,28^. In mainland France, all the measurements conducted in the air, rainwater, and terrestrial products after the 2011 accident showed the absence of significant deposition of radionuclides attributable to the Fukushima accident. The various observations showed that the French regions were affected in a similar way, with spatial and temporal fluctuations due to the movement of air masses. ^137^Cs concentrations were at levels 500 to more than 1000 times lower than those measured at the beginning of May 1986 in France following the Chernobyl accident. In Paris (France), atmospheric fallout deposition from the Fukushima accident was estimated to 2 Bq/m^2^, while ^137^Cs inventories in French soils in 1986 were around 1500 Bq/m^2^^29,30^.

Finally, the industrial use of nuclear energy in France led to additional inputs of ^137^Cs into the environment due to the release of effluent with low level of radioactivity following legal regulations within the atmospheric and aquatic compartments. In rivers, liquid releases are generally not allowed when the flow rates are too low and constrain effluent dilution or when they are too high and enhance the risk of effluent dispersion in case of floodings. Releases along French nuclearized rivers are also performed to limit cumulative effects from nuclear facilities located along a same river. The nuclear industry in France was established in the 1950s and 1960s with the construction of natural uranium graphite gas nuclear reactors (Marcoule and Bugey on the Rhone River, Chinon and Saint-Laurent on the Loire River), a heavy water reactor (Brennilis on the Aulne coastal River) and the first pressurized water reactor in 1967 (Chooz A on the Meuse River). The French nuclear fleet has today 56 operating nuclear reactors spread over 18 Nuclear Power Plants (NPPs), following the shutdown of the two reactors at the Fessenheim NPPs in February and June 2020. Among them, four are located in coastal areas in Northern France while 14 stations are located along 8 large rivers (Loire, Vienne, Rhone, Rhine, Seine, Garonne, Meuse, and Moselle rivers). In 2022, the IAEA reports 438 operational power reactors worldwide and 56 under construction^31^. The French nuclear fleet is the second largest worldwide, after the United States operating 92 nuclear reactors across 54 NPPs.

While the atmospheric monitoring of radionuclides such as ^90^Sr and tritium started at the onset of the military use of nuclear energy in several countries around the world, i.e. in the late 1950s, environmental monitoring in rivers began later although it consisted of sparse measurement stations where only a selection of radionuclides was analyzed. The industrial use of the nuclear energy in France with the rising development of NNPs (pressurized water reactor) in the late 1970s and then the occurrence of atmospheric fallout from the Chernobyl accident in 1986 increased the spatial and temporal patterns of monitoring strategies in river systems.

Riverine sediments keep historical records of numerous particle-reactive trace contaminants, such as long-lived radionuclides, providing precious tools to reconstruct their trajectories over the nuclear era. Nevertheless, alluvial margins are exposed to highly variable sedimentary inputs, both in space and time depending on multiple morphological and hydrological parameters. In this context, the identification of sites with sedimentary archives suitable for reconstructing trajectories of radionuclide contamination over decades represents a major challenge.

In this study, sedimentary archives were collected between 2020 and 2022 in downstream sections of the six largest rivers equipped with nuclear reactors in France (Loire, Rhone, Rhine, Seine, Moselle and Meuse rivers) and were dated using time markers including ^137^Cs, ^241^Am and ^210^Pb_xs_. The combination of these markers may allow to date sediment sequences for up to the last 100 years. They provide for the first time a basis for the reconstruction of radionuclide concentrations and their evolution over time in these major river systems and their export to marine environments over the whole nuclear era. Finally, trajectories of ^137^Cs in riverine sediments are compared to reconstructed atmospheric fallout deposition at the watershed scale to highlight the relative pressure from nuclear industries and the resilience capacity of rivers exposed to this source of contamination.

## Results and discussion

### Dating the sediment cores and their representativeness

**Figure 1:**
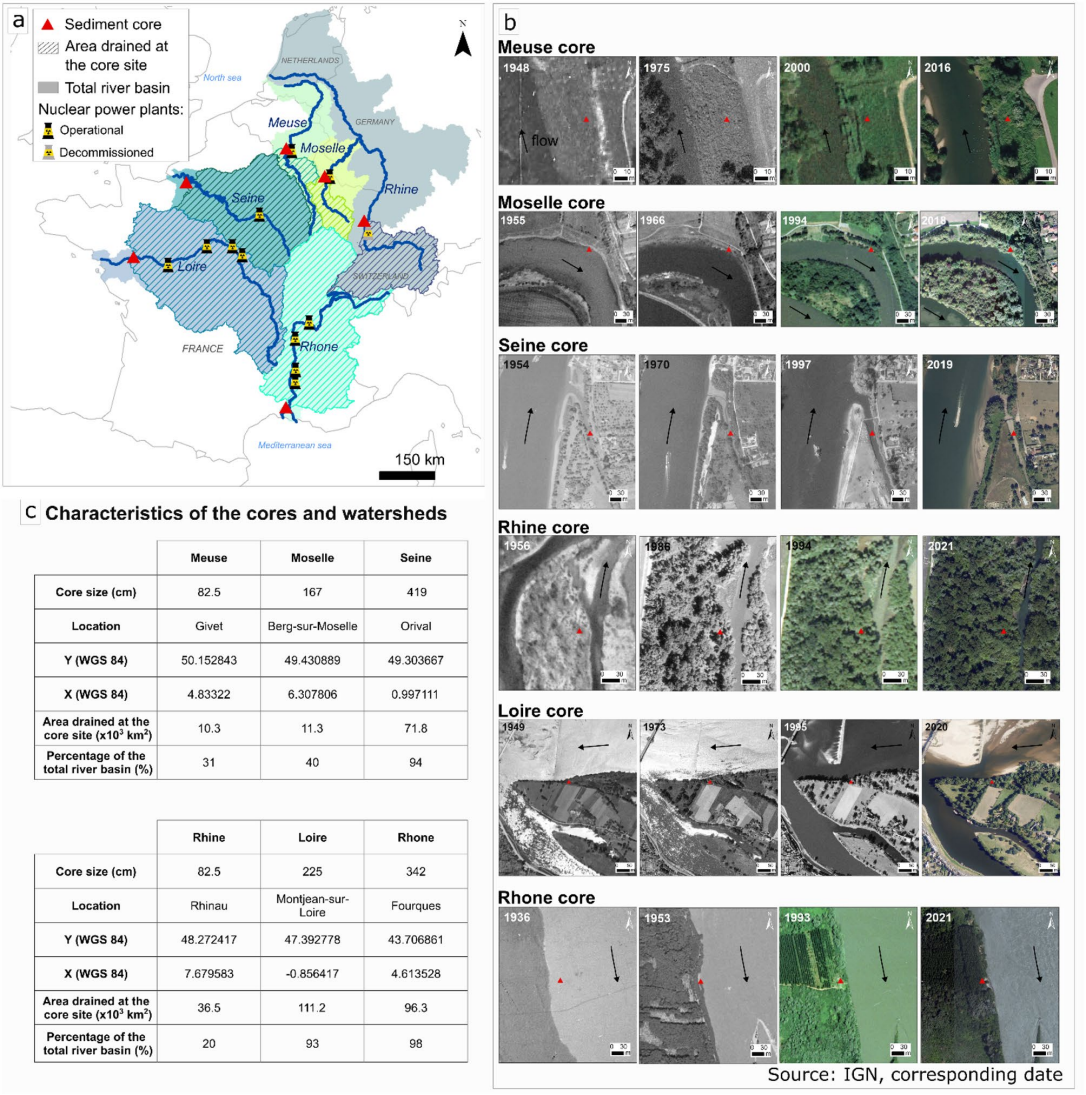
(**a**) Location of Loire, Rhone, Rhine, Seine, Meuse, and Moselle cores with associated drained watersheds (shaded) and location of French NPP’s; (**b**) Historical planimetric evolution of coring sites from aerial photographic archives (photograph source: IGN); (**c**) Characteristics of the cores and watershed. All the cores collected on emerged areas corresponded to sites occupied by riparian forests except the Meuse where grassland vegetation dominates. The Seine core was collected within a continuously submerged secondary channel.

Sediment cores were sampled in downstream sections of the Loire, Rhone, Rhine, Seine, Meuse and Moselle Rivers in France (Fig. 1) by using ancient maps as well as aerial photographs, available literature and field investigations to identify zones where sediment deposition was as continuous as possible over the last several decades. For the Rhone, Meuse and Moselle Rivers several prospective corings, including geophysical survey, were performed to exclude or validate the sites whereas other river sites were selected according to previous works^32–41^. The studied cores were collected at less than 5 m of the main channel, except for the Rhine core collected at 75 m of the main channel and the Seine core, collected in a continuously submerged secondary channel. All the cores collected on emerged areas corresponded to sites occupied by alluvial forest trees and shrubs except the Meuse where yearly vegetation dominates. Geographical contexts are reported in ***SI-1.*** Such concomitant sampling cores on the largest French nuclearized rivers by using a same methodological approach is unique to our knowledge. Sediment core dating is required to determine the trajectories of contaminant concentration over time and challenging, even more when riverine sediment cores collected onto flooding areas are investigated. In contrast with lakes often recording continuous sedimentation, sediment deposition in floodplains may be affected by changes in hydro-sedimentary conditions and site configuration. This means that sedimentation rates most generally vary belong the core accretion, i.e., over time, depending on several parameters such as flooding intensity, duration and occurrence, grain size of deposited particles, geomorphological evolution, and vegetation cover. Sand deposits can lead to increase apparent sedimentation rates (ASR) and additionally dilute most of particle reactive contaminants including ^137^Cs. In this context, riverine sediment core dating requires a rigorous analysis of sedimentary profiles. The combination of chronological tracers such as ^137^Cs, ^210^Pb_xs_ and sandy deposits indicating the occurrence of some historical major floods events is a useful tool to date riverine sediment cores covering the last century^39^. Other dating methods based on persistent organic pollutants (POPs) such as chlorofluorocarbons (CFCs) (e.g., Refs.^42,43^) or trace metals enrichment linked to the rising anthropogenic pressures that characterize the middle of the past century are used as concomitant temporal markers to ^137^Cs and ^210^Pb_xs_ analyses (e.g. Refs.^44–51^). Nevertheless, they are not as accurate due to the lack of precise temporal benchmarks and POP’s are strictly not completely preserved as they do not drop microbial degradation^52^.

**Figure 2:**
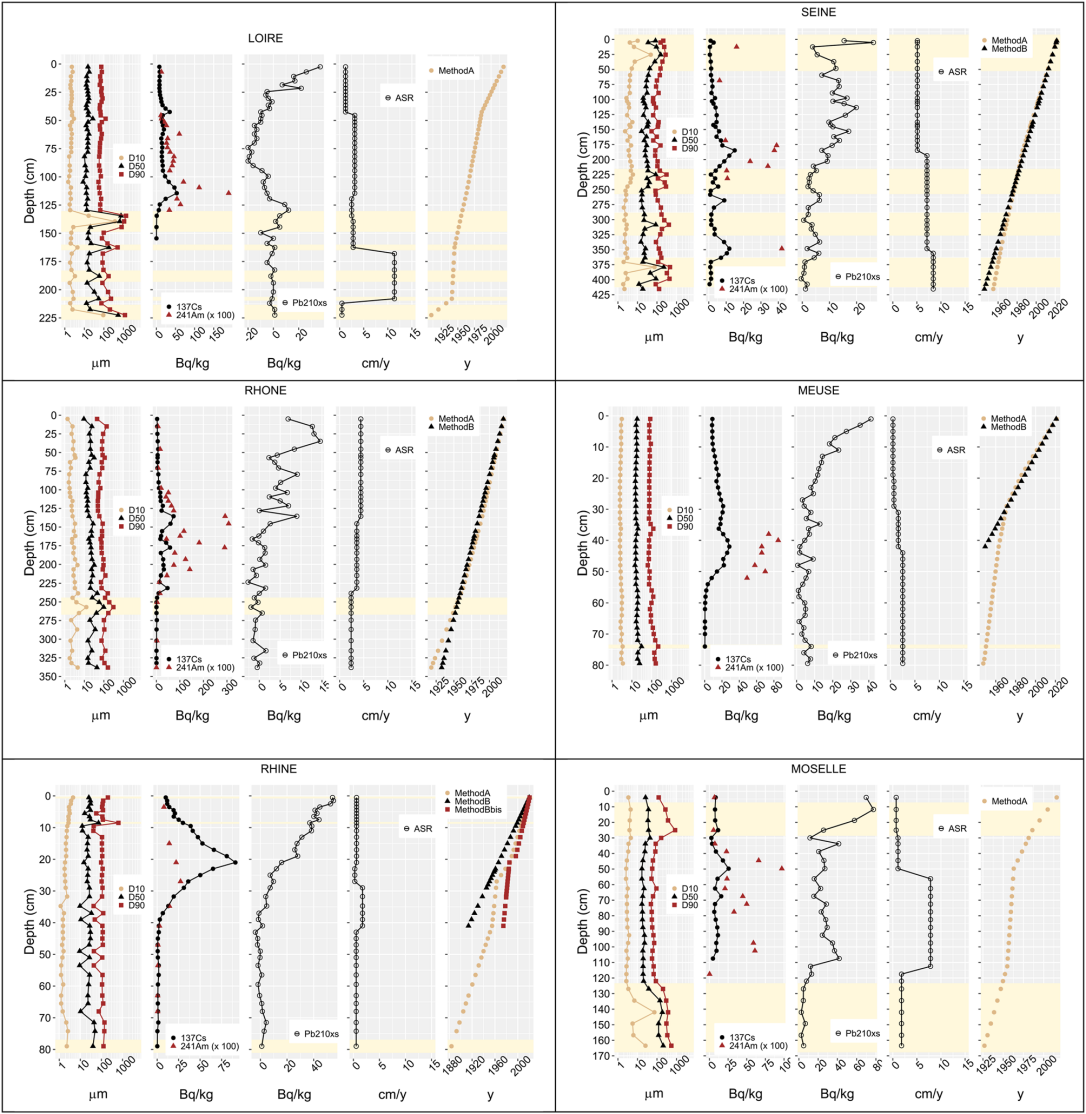
Profiles along the depth (cm) of D10, D50, D90 grain size (µm), ^137^Cs, ^241^Am and ^210^Pb_xs_ (Bq/kg, at the time of sampling), Apparent Sedimentation Rate (ASR, in cm/y) calculated from the Method A (^137^Cs) and estimated age by using either Method A (^137^Cs) or Method B (^210^Pb_xs_)^*^ for the Loire, Rhone, Rhine, Seine, Meuse and Moselle sediment cores; In yellow, sandy strata are shown. ^*^For the Loire and Moselle sediment cores the Method B (^210^Pb_xs_) was not applicable because of the uneven vertical distribution of ^210^Pb_xs_.

Depth profiles (cm) of grain size deciles D10, D50, D90 (µm), measured ^137^Cs, ^241^Am and ^210^Pb_xs_ concentrations (Bq/kg), Apparent Sedimentation Rate (ASR, in cm/y) and estimated mean age model by using Method A (^137^Cs) and Method B (^210^Pb_xs_) are reported in Fig. 2 for the Loire, Rhone, Rhine, Seine, Meuse and Moselle sediment cores. Uncertainties on ASR and consequently on estimated mean age of the sedimentary strata directly depend on the benchmarks deciphered along the depth profile and the thickness of the sedimentary strata where the benchmarks were identified. Uncertainty on estimated mean age mechanically increases as the ratio between ASR and the sedimentary strata thickness decreases. In most of cases, uncertainties on mean age would be less than 3 years by considering that ASR is constant between two successive benchmarks what cannot be strictly testified.

Sediment depth covering the last century ranged from around 80 cm for the Rhine and Meuse rivers until more than 400 cm for the Seine River demonstrating the occurrence of a very wide range of ASR among the studied sites. ASR depends on numerous parameters among which the quantity of sedimentary inputs into the area, flood frequency, deposition conditions, plant cover and sediment bulk density. Several authors report a spatial gradient of deposit thicknesses within alluvial margins (e.g., Refs.^53–55^). This gradient is generally controlled by the distance from the channel and the sedimentation thickness decreases when moving away from the channel^56^. This general gradient can be locally modified by the micro-topography, the nature and density of the riparian forest as well as the type of hydrological connection of the side channels to the main river channels^57^. Reference^58^ showed that for floods with a return frequency of less than 10 years, the banks accumulate sediment and erode beyond that threshold. Reference^59^ specify that it is the filter role played by the riparian forest that controls the sediment retention on the banks. Most generally, there is a grain size gradient which generates a tendency towards the refinement of the granularity of the deposits with increasing distance from the channel. Grain size between and within the cores (D10, D50, D90) remained relatively close except the occurrence of some major sandy strata (D50 > 55 µm or D90 > 175 µm) observed in depth for the Loire River at 134.5–139.5, 162.5, 185–191, 208 and 220–225 cm, the Rhone River at 257 and 338 cm, the Seine River from the surface until 37 cm depth, then at 225, 308.5 and from 379 to 398.5 cm, and the Moselle River between 1.8 and 25 cm depth then below 127 cm (Fig. 2).

**Figure 3:**
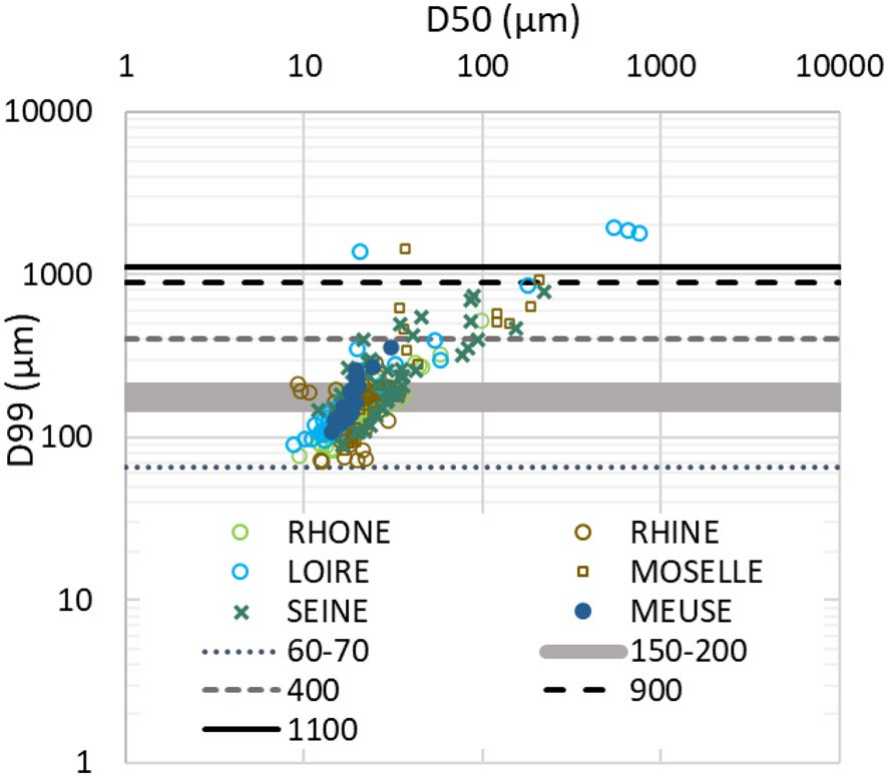
D99 versus D50 grain size (µm) for the Loire, Rhone, Rhine, Seine, Meuse, and Moselle sediment cores, highlighting sediment deposit conditions according to the classification of Ref.^60^; D99 < 60–70 µm: Settling, D99 = 60–70 µm to 150–200 µm: Uniform suspension, D99 =150–200 µm to 400 µm: Graduated suspension, D99 = 400 to 900 µm: Graduated suspension with rolling, D99 = 900 to 1100 µm: Rolling with graduated suspension, D99 > 1100 µm: Rolling.

Apart from the sandiest strata, mean D10, D50 and D90 ranged around 5.6 µm, 35.9 µm and 135.2 µm, respectively, without any significant trends with the depth. Based on the simplified classification of Ref.^60^, our results showed that sediments were mainly deposited by uniform to graduated suspension (60–70 µm < D99 < 400 µm) whatever the studied site, except for the sandy layers deposited from graduated suspension with rolling to rolling (D99 > 400 µm and D99 > 1100 µm) (Fig. 3). Sandy layers are of particular interest in the framework of this study. While sandy particles most generally dilute trace particle reactive contaminants such as artificial radionuclides, sandy deposits may enhance concentrations as well because they originate from erosive events which can remobilize old and poorly solicited contaminated sources. Both induce potential biases on the age model and trajectories if not considered^47^. In contrast, they can be successfully used to identify the occurrence of some major flood events, which can provide additional chronological markers.

The **Loire coring site** was previously studied by several authors (e.g., 32, 33, 34, 35, 36). The ^137^Cs profile on this site displays two major peaks at 42.5 and 114.5 cm depths (34.9 Bq/kg and 53.3 Bq/kg, respectively) whereas it was no longer detected below 134.5 cm. ^241^Am was also found to peak at 114.5 cm depth thereby confirming that this peak corresponds to the maximum atmospheric global fallout from nuclear tests. These ^137^Cs benchmarks were attributed to 1986 (Chernobyl accident), 1963 (Atmospheric global fallout from nuclear tests) and 1955, respectively. The sandy strata found at 134.5–139.5 cm and then at 162.5, 185–191 and 208 cm depths were related to the occurrence of a succession of major floods that occurred, on the one hand, between 1952 and 1955 and, on the other hand, between 1941 and 1945 as suggested by Ref.^35^. The successive sandy layers between 185 and 208 cm depth could also be part a previous major flood event that occurred in 1910, associated in this study to the deepest 5 cm thickness sandy strata at 222.5 cm mean depth. This flood was an exceptional hydrological event, as it lasted for 2 months and contributed 55% of the annual water flow in 1910–1911 as described in Ref.^32^. Owing to these chronological benchmarks, several ASR are determined along the depth. They ranged from 0.5 cm/y for the 1910–1941 period to a maximum of 11.4 cm/y for the 1941–1945 period including the sandy deposits of the floods. Since 1945, ASR showed intermediate values from 3.1 cm/y for the years 1963 to 1986 to 1.2 cm/y for the more recent period (1986–2020) (Fig. 2). As already mentioned in previous works performed on the same coring site, the ^210^Pb_xs_ profile display no excess values mostly due to excess ^226^Ra from unknown origin and cannot be used to cross-check results obtained from method A^35^.

**The Rhone core** showed increasing ^137^Cs activities starting from 250.5 cm depth, which can be attributed to 1955 (Fig. 2). Activities of ^137^Cs and ^241^Am vary towards maximum values ranging from 47.3 to 73.2 Bq/kg between 233 and 139 cm highlighting the chronical releases from the spent fuel reprocessing plant (Marcoule). Those industrial releases culminated over the 1963–1990 period and partially masked the Chernobyl accident contribution^61,62^. By using these chronological benchmarks, estimated ASR amount to 2.4, 3.6 and 4.5 cm/y for the periods 1955–1963, 1963–1990 and 1990–2020, respectively. These values are very close to that calculated over the whole 1930–2020 period by using the ^210^Pb_xs_ method, i.e., 3.8 cm/y. Therefore, a mean apparent sedimentation rate of 3.8 cm/y has been extrapolated to the period before 1955.

Our results showed that along the **Rhine sedimentary core**, ^137^Cs mainly peaks at 21 cm depth (91.1 Bq/kg) while ^241^Am peaks at around 27 cm (Fig. 2). These maxima are attributed to 1986 and 1963, respectively, whereas the drastic ^137^Cs concentration decrease towards detection limits below 41 cm indicates that the pre-bomb test period was reached in 1955.^39^ indicate that the fine grained sandy strata at 75-80 cm depth (D90 ranging from 125 to 139 µm) probably corresponds to extreme flood events, after the beginning of the first phase of the engineering works during the nineteenth century^63^, such as the Q ≥ 300 floods of 1876 with a maximum mean daily discharge (Qmd.max) of 5,530 m^3^/s and/or the floods of 1881 (Qmd.max = 4,764 m^3^/s, ~ Q200) and 1882 (Qmd.max = 4,371 m^3^/s, ~ Q70) and the authors finally attributed to 1876–1882 the benchmark at 80 cm. By using all these chronological tracers (Method A), calculated ASR are 0.5, 1.8, 0.3 and 0.6 cm/y for the 1882–1955, 1955–1963, 1963–1986 and 1986–2021 periods, respectively, which is in line with those estimated by using the ^210^Pb_xs_ approach. In the case of the Rhine core, method B was applied by considering first a single ASR characterizing the whole depth from the surface to 41 cm depth where negligible ^210^Pb_xs_ contents are recorded, and then by considering two ASR associated with the two apparent sections from the surface to 19 cm and from 19 to 41 cm depth. These last approaches provide estimated ASR of 0.4 cm/y in the first case and of 0.8 and 1.9 cm/y by considering the two segments. Figure 2 shows that these last ASR values match the ASR calculated by method A. The rather low ASR characterizing the Rhine core reflect both the hydrodynamic condition specific to the site and the significant distance from the main channel of the Rhine River. The absence of clear ^137^Cs peak at 27 cm associated to 1963 would indicate either a partial masking due to industrial releases or a smoothing of ^137^Cs concentration owing to the rather small ASR (0.3 cm/y) compared to the thickness of the strata sampled (2 cm). Swiss NPP’s releases started in 1968 and may have significantly affected ^137^Cs concentration in riverine sediments as already observed by^64^.

The **Seine core** displays undetectable ^137^Cs at the bottom of the profile, i.e., at 415.4 cm depth (< 0.1 Bq/kg) (Fig. 2), which means that the pre-bomb test period is reached. The low grain size of this strata (D50 = 18.7 µm) excludes a potential dilution effect. Even though disrupted by several sandy strata, the core displays two main ^137^Cs peaks at 184.8 and 348.3 cm (14.9 and 11.8 Bq/kg, respectively) which are attributed to 1986 and 1963, respectively. Based on these three chronological benchmarks, estimated ASR amount to 8.4, 7.1 and 5.1 cm/y for the periods 1955–1963, 1963–1986 and 1986–2022, respectively. The ^241^Am is detected for the 1^st^ time at 343.3 cm and is concomitant to the ^137^Cs peak attributed to 1963 then significant varying contents are found towards the surface until 68.5 cm. The lowest contents are observed for the sandiest strata and can be explained by dilution effects. The detection of ^241^Am all along the profile most probably sign the releases from nuclear installations located upstream, namely in the Paris city area. Among them, the Fontenay-aux-Roses center early started research devoted to military then civilian uses of the nuclear energy in the late 1950s and was authorized to release radioactive effluents into the Seine River for several decades. The detection of ^241^Am at various depths could also originate from the liquid releases of the spent nuclear fuel reprocessing plant located in La Hague (France) along the Manche cost due to particles rebounds during large tides affecting the Seine River ^65^. The method B confirms the method A with a mean ASR of 5.5 cm/y over the period 1946–2022 allowing to establish the age/depth model (Fig. 2). Finally, referring to the main floods (> 2000 m^3^/s) recorded at the Poses hydrographic station located around 25 km upstream from the coring site, the significant sandy strata observed at 225, 308.5 and from 379 to 398.5 cm, attributed to the years 1980.3, 1968.6 and 1957.0 to 1959.3 with uncertainties corresponding to a couple of years could originate from the deposits of the major floods that occurred on 01/16/1982, 03/06/1970 and 01/24/1955, respectively.

Four benchmarks are retained for the **Meuse core** at 62, 42, 29 and 19 cm depth for the first detection of ^137^Cs (1955) and the three characteristic ^137^Cs peaks. The ^137^Cs peak at 29 cm depth is attributed to the accidental release from the Chooz NPP’s that occurred in 1971 while the two others are associated with the years 1963 and 1986. In 1971, the ^137^Cs annual releases from the Chooz NPP’s were about tenfold higher than those that occurred in previous and following years, which explains the detection of this atypical peak. The detection of ^241^Am close to the ^137^Cs peak at 42 cm depth attributed to 1963 confirms the origin of this peak. Based on these chronological benchmarks, ASR are estimated to 2.5, 1.6, 0.7 and 0.5 cm/y for the successive periods of 1955–1963, 1963–1971, 1971–1986 and 1986–2021, respectively. Method B applied from the surface to 42 cm depth where stable ^210^Pb_xs_ contents are reached provides an ASR estimation of 0.6 cm/y in close agreement with the values determined by using the method A.

Rising ^137^Cs activities start at 112.5 cm depth for the **Moselle core** in fine grain sediments deposited above the 37 cm thick sandy strata observed in the deepest layers of the core, i.e., below 127 cm depth. Below 112.5 cm ^137^Cs concentrations were below the limit of detection (0.2 Bq/kg). The layer at 112.5 cm depth is then associated to 1955 while the ^137^Cs peaks corresponding to the global atmospheric fallout from nuclear tests and the Chernobyl accident are found at 49.8 and 25 cm depth, respectively. Disrupted profiles of both ^137^Cs, ^241^Am and ^210^Pb_xs_ between 112.5 and 30 cm depth most probably highlight the occurrence of a discontinued sedimentation due to either a partial remobilization of previously deposited sediments before bank stabilization or sediment inputs from varying origins. The emergence of the “Ile aux Oiseaux” island in the late 1950s due to the Moselle channelization to protect from erosion the Saint Michel church overhanging the steep valley side at Berg-sur-Moselle could explain this disruption (**SI-1**). Finally, reconstruction of floods made from data recorded at the nearby Uckange hydrometric station, located around 35 km upstream of the coring site, in operation since 1981, and archive water heights data for the previous period indicates the occurrence of an extreme flood in 1947. The thick sandy layer below 127 cm was attributed to this major hydrological event. From these chronological benchmarks, ASR of 1.8, 7.8, 1.1 and 0.7 cm/y are associated to the successive periods 1947–1955, 1955–1963, 1963–1986 and 1986-2021. The method B would confirm the chronology of the core with a mean ASR of 1.7 cm/y for the whole period 1947–2021.

Owing to the various ASR calculated for each core by using the Method A, age/depth models are obtained and used to attribute years to successive sediment layers. Those ages are associated with the mean depth of the layers of various thicknesses. Minimum and maximum ages were calculated by using the maximum and minimum depths of each layer, respectively, and would represent absolute uncertainties on ages. All the data are compiled in SI-Table 1**.** From these dating results, trajectories of ^137^Cs concentrations over the last decades can be drawn and compared to atmospheric fallout deposition on the catchments.

### Atmospheric fallout on the catchments over the last decades

**Figure 4:**
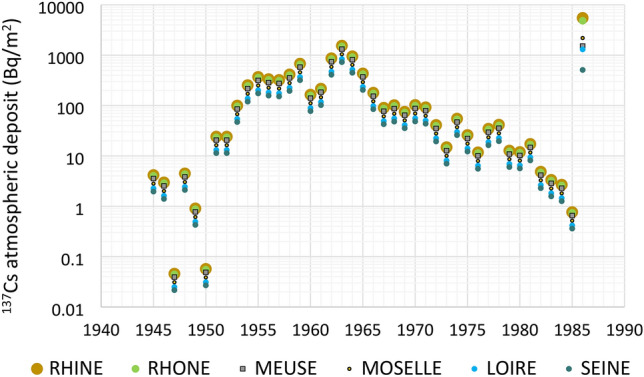
Atmospheric deposits of ^137^Cs (Bq/m^2^) reconstructed from Ref.^26^ over the period 1945–1986 for the Loire, Meuse, Moselle, Rhine, Rhone and Seine catchments upstream the core sampling sites.

**Figure 5:**
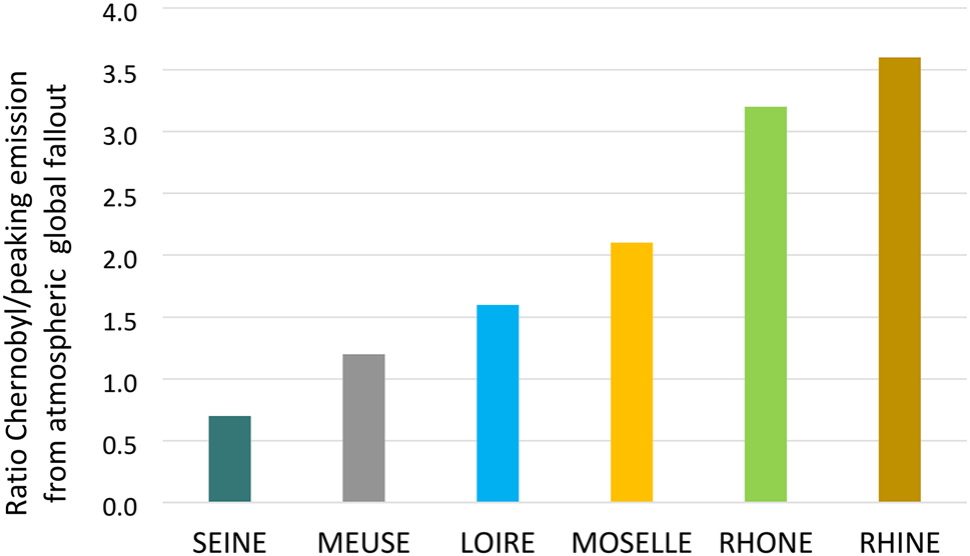
Ratio between ^137^Cs deposits from atmospheric fallout from the Chernobyl accident and those from peaking global fallout from nuclear tests in 1963 for the various catchments.

The reconstruction of mean ^137^Cs atmospheric fallout onto the studied catchments over the period 1945–1986 shows a peak in deposition in 1963, with soil inventories ranging from 729 to 1544 Bq/m^2^ and a progressive decrease until the mid- 1980s when deposition became fully negligible (0.1 Bq/m^2^) (Fig. 4). ^137^Cs deposition from the global atmospheric fallout did not vary strongly among the studied catchments even though the Rhine and the Rhone were the most impacted with an annual fallout in 1963 close to 1500 Bq/m^2^. This same year, the Seine and the Loire catchments received the lowest atmospheric deposition (729 and 840 Bq/m^2^, respectively) while the Moselle and the Meuse displayed intermediate values (1045 and 1320 Bq/m^2^, respectively) (Table 1). Global atmospheric fallout from nuclear tests led to a long-lasting contamination of the atmosphere, soils, and river systems for the following decades. In 1986, atmospheric fallout from the Chernobyl accident led to the re-increase of the baseline of atmospheric deposition by a factor 1000 to 10,000 (Fig. 4). At that time, the Rhine, Rhone, Moselle and Meuse catchments received their highest annual deposits, estimated to 5489, 4825, 2192 and 1520 Bq/m^2^, respectively. These atmospheric depositions were even almost four-fold higher than those received in 1963 in the case of the Rhine catchment (Fig. 5). The Seine and Loire catchments were the least exposed with atmospheric fallout of 510 and 1305 Bq/m^2^, respectively, these values remaining close to those received during the peak in atmospheric global fallout in 1963. At the scale of the studied catchments, the spatial heterogeneity of the global atmospheric fallout is mainly due to meteorological factors controlling the deposition of radionuclides scavenged from the atmosphere together with precipitation or in dry form. Dry atmospheric fallout of radionuclides is the most uniform and this uniformity increases with the distance from the source of emission^66,67^. In contrast, wet deposition of radionuclides, mainly driven by rainfall and snowfall, is strongly heterogeneous in space. These latter parameters mainly drove the atmospheric fallout from the Chernobyl accident, together with the pattern of air masses circulation at the scale of Europe during the days that followed the event^25,26,68,69^. Furthermore, as radionuclides from the Chernobyl accident were emitted in lower atmospheric layers (i.e. troposphere) compared to those from the nuclear tests (i.e. stratosphere), the Chernobyl radiocaesium deposition was much more heterogeneous across space than the global fallout, because it originated from few distinct precipitation events that occurred late in April and early in May 1986, when the radioactive cloud travelled across the European continent^26^.

**Table 1:** Atmospheric deposits of ^137^Cs (Bq/m^2^) reconstructed from Ref.^26^ over the period 1945–1986 for the Loire, Meuse, Moselle, Rhine, Rhone, and Seine catchments upstream the core sampling sites.

	LOIRE	MEUSE	MOSELLE	RHINE	RHONE	SEINE
1945	2	4	3	4	4	2
1946	2	3	2	3	3	1
1947	0	0	0	0	0	0
1948	2	4	3	4	4	2
1949	0	1	1	1	1	0
1950	0	0	0	0	0	0
1951	13	21	16	24	23	11
1952	13	21	16	24	23	11
1953	54	85	68	100	97	47
1954	138	217	172	254	246	120
1955	201	316	250	370	358	175
1956	182	286	226	334	324	158
1957	175	275	218	322	312	152
1958	225	354	280	414	401	195
1959	368	579	458	677	656	320
1960	89	140	111	164	159	78
1961	117	185	146	216	209	102
1962	471	741	586	867	840	409
**1963**	**840**	**1320**	**1045**	**1544**	**1496**	**729**
1964	517	812	643	950	920	448
1965	236	371	293	433	420	205
1966	98	154	122	180	174	85
1967	49	77	61	90	87	43
1968	55	87	69	102	99	48
1969	41	64	51	75	73	36
1970	56	88	69	103	99	48
1971	50	79	62	92	90	44
1972	22	35	28	41	40	19
1973	8	13	10	15	14	7
1974	30	47	37	55	53	26
1975	14	22	18	26	25	12
1976	6	10	8	12	11	6
1977	19	30	23	35	33	16
1978	23	36	28	42	40	20
1979	7	11	9	13	12	6
1980	6	10	8	12	12	6
1981	9	15	12	17	17	8
1982	3	4	3	5	5	2
1983	2	3	2	3	3	2
1984	1	2	2	3	3	1
1985	0	1	1	1	1	0
**1986**	**1305**	**1520**	**2192**	**5489**	**4825**	**510**
1987	0	0	0	0	0	0
1988	0	0	0	0	0	0

Significant values associated to peaking atmospheric deposits in 1963 and 1986 are in bold.

### Temporal trajectories of ^137^Cs concentration in riverine sediments over the last decades

**Figure 6:**
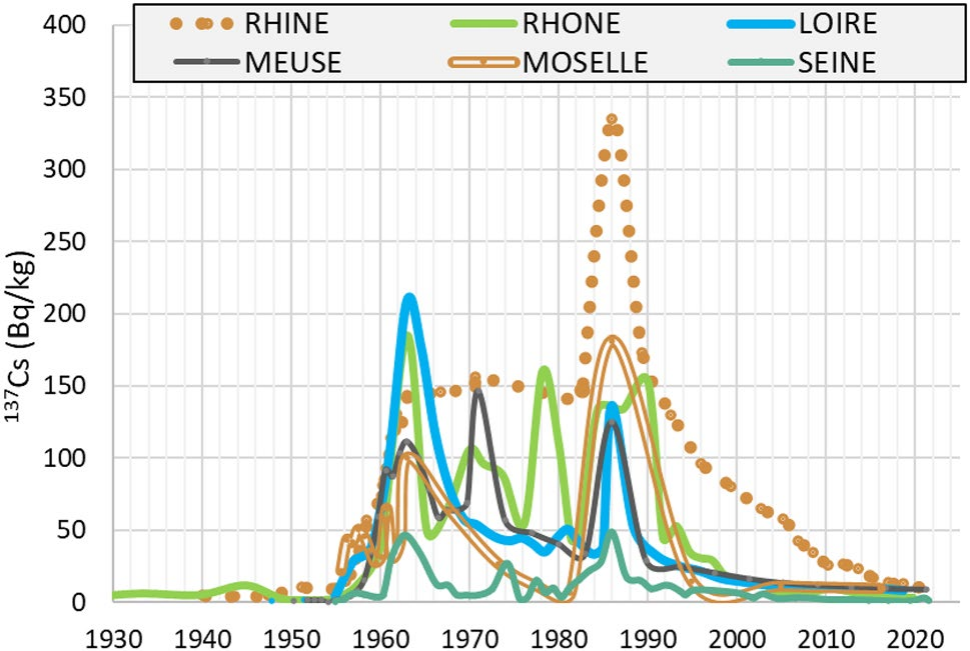
Temporal trajectories of ^137^Cs concentration in sediments of the Loire, Rhone, Rhine, Seine, Meuse and Moselle rivers over the nuclear era, decay-corrected to the sampling date (initially deposited).

As ^137^Cs trajectories are decay-corrected to the sampling date (Fig. 6), its concentrations (Bq/kg) reported on this graph correspond to those prevailing when sediment deposited. Furthermore, ^137^Cs peaks associated with Chernobyl fallout integrate varying time periods depending on both ASR and the thickness of the corresponding sediment layer. These Integrated Periods (IP, SI-Table 1) vary over a wide range, i.e., from 1.4 y for the Seine core to 6.8 y for the Moselle core, leading to potential significant underestimation (i.e., dilution) of ^137^Cs concentrations associated with this accidental source of contamination. To avoid such biases, ^137^Cs concentrations associated with the Chernobyl peak were corrected from dilution by multiplying for each core ^137^Cs deposited concentrations to associated integrated period used here as dilution coefficient. The higher the integrated period the smoother the Chernobyl peak, which is then underestimated. Such correction was obviously not applied to peaking emission from the global fallout in 1963 since this source of contamination took place for several years in contrast with the Chernobyl event. In contrast, a correction for integrated period dilution was applied in the case of the Meuse for the year 1971 when a sharp industrial release was performed by the Chooz NPP’s (DIRATA data base). Once corrected from peak dilution by integrated period, representative ^137^Cs trajectories in riverine sediments can finally be drawn for each river to be compared. ^137^Cs trajectories obtained for the nuclearized French rivers display very different shapes and trends over time. Those reflect the responses of riverine sediments to multiple sources of radioactive contamination including long-lasting or accidental atmospheric fallout of ^137^Cs across the catchments and regulatory liquid releases from the nuclear industries located along the river.

**Figure 7:**
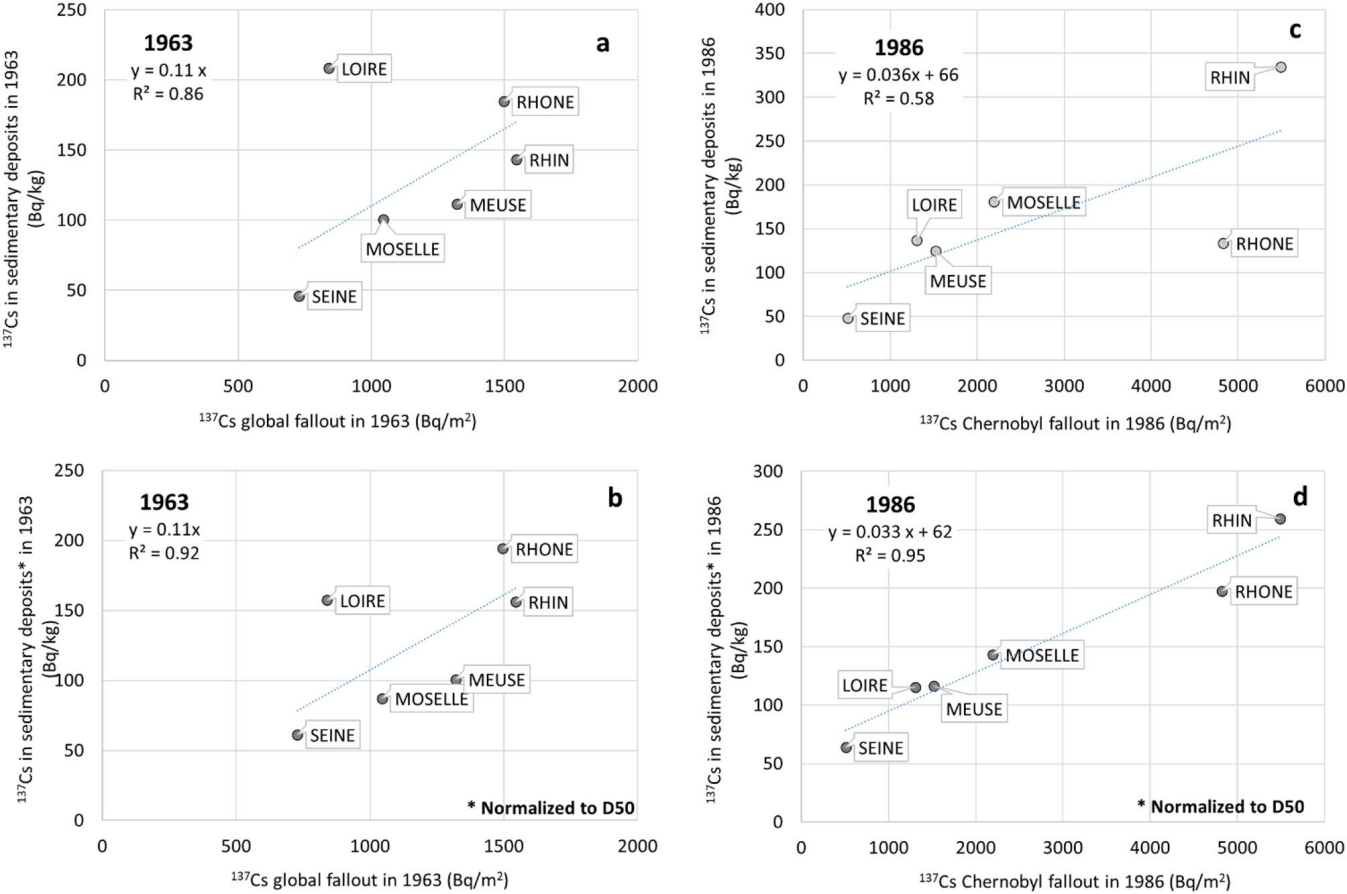
^137^Cs concentration (Bq/kg) in sedimentary deposits following global fallout from nuclear tests peaking in 1963 (**a**) and Chernobyl accident in 1986 (**c**) versus ^137^Cs inventories (Bq/m^2^) reconstructed for the same years for the Loire, Rhone, Rhine, Seine, Meuse, and Moselle rivers; (**b**) and (**d**) report ^137^Cs concentrations in sediments once normalized to D50 grain size.

The contribution of the global atmospheric fallout appears maximal for the Loire, Rhone, and Rhine rivers, with ^137^Cs concentrations in sediments at the time of their deposition, reaching 208 Bq/kg (for the Loire River), while the Seine River displays the lowest contents, i.e., ca 50 Bq/kg. The Meuse and Moselle rivers show intermediate values even though, in the particular case of the Moselle core, ^137^Cs concentrations might have been under-estimated due to the contribution of industrial sludges (estimated at 30% more or less)^70,71^. The correlation between ^137^Cs concentrations in sediments deposited in 1963 and atmospheric fallout across their respective catchment is significant (R^2^ = 0.86), except for the Loire which discards from the linear relation (Fig. 7a). The D50 of the Loire sediments deposited in 1963 is particularly low (< 15 µm) when compared to 1963 sedimentary strata from the other cores (mean D50 > 25 µm). This may explain at least partly the higher ^137^Cs contents observed in the Loire sediment layer attributed to 1963 when compared the 1963 referential values for this river estimated from the relationships reported on Fig. 7a. Normalizing ^137^Cs concentrations to grain size improved the relationships (R^2^ = 0.92, Fig. 7b).

Chernobyl contamination of riverine sediments ranged in 1986 from 48.5 Bq/kg for the Seine River to almost 335 Bq/kg for the Rhine River. This same year, ^137^Cs contents in sediments of the Loire, Meuse, Rhone and Moselle rivers remained close one from each other, and ranged from 125 to 180 Bq/kg. Those levels are in rather good agreement with the levels of atmospheric fallout from the accident on the catchments (Fig. 7c). Correlations between ^137^Cs contents in sediments and ^137^Cs atmospheric fallout are improved when taking the grain size of sediments into account with R^2^ rising from 0.58 to 0.95 (Fig. 7d). These results likely highlight that the studied river systems display a similar behavior in terms of soil erosion and sedimentary dynamics as they responded similarly to atmospheric contamination. Downstream of large rivers, morphoclimatic parameters characterizing catchments such as landscape, geology, soil occupation, climate and sediment dynamics would be sufficiently homogeneous at this scale (> 10 10^3^ km^2^) to induce comparable contamination levels of riverine sediment originating from atmospheric fallout deposition of particle reactive trace elements such as ^137^Cs on the catchment. Relationships obtained between fine grained riverine sediment contamination and atmospheric fallout deposition on the catchment either from chronic deposition or accidental events provide useful tools in the frame of risk assessment and to predict river water contamination levels in the context of accidental ^137^Cs atmospheric fallout deposition. Referring to the mean residual ^137^Cs contents in riverine sediments from nuclear tests at the beginning of the 1980s (62 Bq/kg, Fig. 7d), atmospheric fallout from the Chernobyl accident was almost negligible for the Seine basins while those increased by a factor 5 ^137^Cs concentrations in sediments of the Rhine River. Apart from atmospheric fallout, evidence of significant imprints from industrial radioactive releases are observed for the Rhone, Meuse and Rhine rivers over the 1970–1990 period (Fig. 6). ^137^Cs contamination of the Loire, Moselle and Seine rivers was dominated by atmospheric fallout deposition on their catchments. Finally, in the case of the Moselle, the contribution of industrial sludges necessarily reduced the proportion of detrital particles carrying ^137^Cs because steel particles are not exposed to atmospheric fallout like soil particles and do not easily fix ^137^Cs released by the nuclear industries.

## Conclusions

Sediment cores collected from 2020 to 2022 in the downstream sections of the six major nuclearized large rivers in France allowed to reconstruct ^137^Cs temporal trajectories over the whole nuclear era. These trajectories cover almost 75% of the metropolitan French territory. The studied coring sites displayed varying apparent sedimentation rates either with the depth or between the sites (0.4–12 cm/y). They were precisely determined thanks to the chronological radioactive tracers used, namely ^137^Cs and ^210^Pb_xs_, coupled with historical extreme flooding benchmarks. Selected riverbank sites, except for the Seine, generally exhibited similar particle grain sizes, primarily deposited by uniform to graduate suspension, besides to flood event deposits. The reconstruction of ^137^Cs atmospheric fallout deposition at the catchment scale was used to validate temporal ^137^Cs trajectories in riverine sediments.

The contribution of the global atmospheric fallout from nuclear tests to ^137^Cs contamination of riverine sediments was maximal for the Rhone, Loire, and Rhine rivers while the Seine River displays the lowest contents. Atmospheric fallout from the Chernobyl accident most significantly increased ^137^Cs contamination in sediments of the Rhine > Rhone > Moselle rivers and was almost negligible for the Seine River. Evidence of significant imprints from industrial radioactive releases were observed for the Rhone, Meuse and Rhine rivers over the 1970–1990 period. The studied river systems display over all rather similar responses to atmospheric contamination, i.e. soil erosion and sedimentary dynamics. Seventy years after the first introduction of artificial radionuclide ^137^Cs in the environment and although the wide use of the nuclear combustible in industry, French riverine fine-grain sediments today typically contain few Bq/kg, which is approximately ten time the detection limits.

## Materials and methods

### Sediment sampling and core dating

#### Sampling

Sediment cores were collected between 2020 and 2022 on the Loire, Rhone, Rhin, Seine, Meuse and Moselle rivers by using a percussion driller (Cobra TT, SDEC, France) with transparent PVC tubes (diameter 46 mm or 100 mm). For cores exceeding 1-m depth, a master core was obtained by sampling twice successive 1-m sediment cores across less than 1 m^2^ surface area. The second coring was vertically shifted by 50 cm compared to the first one to preserve sediment from interface disruptions. Once back to the laboratory, each 1-m core was longitudinally cut and open for stratigraphic analyses, and then laterally cut. From 1 to 16.5 cm sediment slices were successively sampled from the surface to the depth depending on the visual observation of core stratigraphy. Slices were stored at − 25 °C and freeze-dried under dehydrated nitrogen flux to avoid any atmospheric exchange and sieved to 2 mm before further analyses.

#### ^137^Cs and other chronological tracers dating method (method A)

Sediment core samples were dated by using ^137^Cs initially introduced into the atmosphere during the atmospheric nuclear testing performed all around the world between 1945 and 1980. For several countries, atmospheric testing was banned in 1964 even though France and China did not sign immediately the Test Ban Treaty and stopped atmospheric testing in 1974 and 1980, respectively. The ^137^Cs dating approach consists in identifying a layer enriched in ^137^Cs in the sediment cores, which is attributed to the peak of atmospheric fallout. This approach allows assessing sedimentary deposition ages assuming that subsequent vertical migration due to diffusion or bioturbation mechanisms are negligible. In addition, the lack of detection of ^137^Cs in depth may indicate that sediment deposited before the atmospheric bomb testing with fallout detectable at the global scale, i.e. before 1954^7^. Artificial alpha emitters radionuclides such as ^238^Pu, ^239,240^Pu, ^241^Am and ^244^Cm may provide additional chronological tracers. Their emission also peaked in the environment in 1963 due to global atmospheric fallout from nuclear tests although they were also released through respecting the regulations in the Rhone River by the Marcoule spent nuclear fuel reprocessing plant mainly from 1964 to 1990 when this facility started to be dismantled for several decades. Chronological tracers identified with depth in sediment cores allowed to calculate mean Apparent Sedimentation Rates (ASR) between two successive benchmarks. Then, the age of individual sediment layers was determined by considering that the sedimentation rates are constant over the different periods. Uncertainties associated with the age can be estimated from potential mismatches on ASR and the thickness of sediment slices. In this study, they ranged from < 1 year for the Rhine core to ± 3 years for the Rhone core.

#### ^210^Pb_xs_ dating method (method B)

Sediment cores were also dated by using Lead-210 isotope in excess (^210^Pb_xs_) as a daughter radionuclide of the ^238^U radioactive decay series produced by gaseous ^222^Rn decay. The ^210^Pb_xs_ reaches soils through dry and wet deposition from the atmosphere and is widely used to date lake sediment where atmospheric inputs are continuous^7,72^. By considering the ^210^Pb_xs_ flux to be constant, the apparent sedimentary rate can be estimated from the linear regression of ln ^210^Pb_xs_ versus depth^73^, according to Eq. (1):

1 $${ln({}{}^{210}Pb}_{xs})={ln({}{}^{210}Pb}_{0 xs})-(\lambda \frac{x}{v})$$

With ^210^Pb_0xs_ the initial ^210^Pb_xs_ at the time of deposition, *λ* the radioactive decay constant of ^210^Pb (0.0311 y),* x* the sediment depth relative to the surface (e.g. cm) and *v* = *x/t,* the sedimentation rate (e.g. cm/year). In a system where the initial ^210^Pb_xs_ is constant over time, Eq. (2) shows that depth variation of ^210^Pb_xs_ in the sediment column provides the sedimentation rate. This approach requires measuring the activity of ^210^Pb and of one of its ascendants in radioactive equilibrium with ^226^Ra. In the present case, as classically done, the chosen nuclide is ^214^Bi. The ^210^Pb_xs_ dating method applied to sedimentary archives collected on alluvial margins is only used here in this study to validate method A because this approach can overcome several biases when applied to such sedimentary systems due to (i) variation of sedimentary fluxes over time, (ii) partial remobilization of sedimentary deposits during flooding, (iii) atmospheric contamination and (iv) variation with time of the origin of the sedimentary masses.

Both methods A and B are widely used to date recent sediment deposits (100 last years) because ^137^Cs and ^210^Pb half-life (30.08 y and 22.20 y, respectively) allow to cover several decades of sediment deposition^73^.

### Low-background gamma spectrometry

Sedimentary core samples were analyzed by gamma spectrometry. Dry samples were conditioned in 17- or 60-mL tightly closed plastic boxes for gamma counting using low-background and high-resolution Germanium Hyper pure detectors at the IRSN/LMRE laboratory in Orsay^74^. The boxes were placed in vacuum-sealed packages and stored during at least one month before measurement to ensure the secular equilibrium of the ^210^Pb necessary to determine the concentration of ^210^Pb_xs_. Efficiency calibrations were constructed using gamma-ray sources in a 1.15 g/cm^3^ density solid resin–water equivalent matrix. Activity results were corrected for true coincidence summing and self-absorption effects^75^. Measured activities, expressed in Bq/kg dry weight were decay-corrected to the date of sampling. The activity uncertainty was estimated as the combination of calibration uncertainties, counting statistics, and summing and self-absorption correction uncertainties. A wide range of gamma emissions are detected with a germanium detector including ^137^Cs, ^210^Pb and ^214^Bi used to determine ^210^Pb_xs_.

### Sediment grain size distribution

The grain size distribution was analyzed by laser diffraction (MASTERSIZER hydro2000G, laser wavelength) on the samples, previously homogenized and moistened if needed. The grain size is calculated from the pattern of the scattered light produced by a dispersed system of particles when the laser beam passes through it. The relationship between particle size and distribution pattern of light is provided by two approximation modes: the Fraunhofer diffraction theory and Mie scattering theory. Grain size was measured on the range 0.02–2000 µm and statistics such as the median grain size (D50) were calculated using GRADISTAT v8.0 such as D10 and D90^76^; with D10, D50 and D90 the particle grain size (µm) for which 10, 50 and 90% of the volume percentage of particles, respectively, are lower.

### Reconstruction of radiocaesium atmospheric fallout

Reconstruction of ^137^Cs atmospheric fallout was carried out with the R software and the following packages: sf^77^, ggplot2^78^, raster^79^, rgdal^80^, leaflet^81^ and dplyr^82^.

Published open accessed baseline maps on atmospheric nuclear weapon tests- and Chernobyl- derived ^137^Cs inventories (Bq/m^2^) in soils^26^ were used to reconstruct the annual deposition of ^137^Cs (1945 – 1993) in the investigated catchments. Briefly, ^239+240^Pu and ^137^Cs activities were measured in undisturbed topsoil samples (0–20 cm) collected in Western Europe in the framework of the Land Use/Cover Area frame survey (LUCAS). Generalized additive models (GAM) with environmental factors were used to predict, with a spatial resolution of 500 m, ^137^Cs and ^239+240^Pu inventories (decay-corrected to the 1August 2009). Importantly, areas above 1000 m altitude were masked. Data from^2^ regarding the annual deposition of radionuclides (PBq) in the Northern hemisphere following atmospheric nuclear testing was used to reconstruct the annual percentage deposition of ^137^Cs in the 40–50° North latitudinal band, assuming an area of 31.5 × 10^12^ m^2^ and a fractional deposition of 0.0221. Based on this fallout chronicle, the annual proportion of ^137^Cs fallout (and corresponding inventories decay-corrected to 2009) was calculated by using the baseline maps from^26^. Every pixel value (i.e. ^137^Cs inventory) created with this method was decay-corrected according to Eq. (2).

2 $${}^{137}{\text{Cs}}_{i}, Y (\text{Bq}/\text{m}2)\hspace{0.17em}=\hspace{0.17em}{}^{137}{\text{Cs}}_{i}, 2009 (\text{Bq}/\text{m}2) {e}^{(-\uplambda *(2009-Y))}$$

Where ${}^{137}{Cs}_{i}$*, Y (Bq/m*^*2*^*)* is the corrected ^137^Cs inventory for pixel i, Y is the year ranging from 1945 to 1993, ^*137*^*Cs*_*i*_*, 2009 (Bq/m*^*2*^*)* is the predicted ^137^Cs inventory for pixel i by^26^ and λ is the decay constant of ^137^Cs (λ = Ln2/30.1 y). Similarly, Eq. (2) was used to reconstruct the ^137^Cs inventories in 1986 following the Chernobyl accident. Considering the presence of point anomalies in the initial Chernobyl derived fallout map used in this study, values above 60 kBq/m^2^ have been masked (which correspond to the second highest value measured in the LUCAS samples). Finally, the *cellStats* function from the *raster* package was used to calculate the mean annual deposition of ^137^Cs between 1945 and 1993 for each catchment.

### Industrial releases

Radiocaesium discharges from the nuclear industries located upstream of the coring sites were extracted from the International Atomic Energy Agency (IAEA) database on Discharges of Radionuclides to the Atmosphere and the Aquatic Environment (DIRATA). This database contains information on the discharges of radionuclides into the environment from nuclear facilities around the world.

### Supplementary Information

Supplementary Information.

## Data Availability

All data generated or analyzed during this study are included in this published article [and its supplementary information files].
