# Supplementary material for: Temporal trajectories of artificial radiocaesium 137Cs in French rivers over the nuclear era reconstructed from sediment cores
Source: Sci Rep. 2024 Jun 20;14:14213. doi: 10.1038/s41598-024-64505-7 (PMC11189920; doi:10.1038/s41598-024-64505-7)
Supplement: Supplementary file 1 — Supplementary Information. [file 41598_2024_64505_MOESM1_ESM.docx]

**Temporal trajectories of artificial radiocaesium ^137^Cs in French rivers over the nuclear era reconstructed from sediment cores**

EYROLLE Frédérique^1*^, CHABOCHE Pierre-Alexis^2,3^, LEPAGE Hugo^1^, NICOULAUD GOUIN Valérie^1^, BOYER Patrick^1^, DE VISMES Anne^4^, SEIGNEMARTIN Gabrielle^5^, BADARIOTTI Dominique^6^, CHABAUX François^7^, CHASTANET Maxime^8^, CLAVAL David^1^, COPARD Yoann^9^, COYNEL Alexandra^8^, DEBRET Maxime^9^, DELUS Claire^10^, EUZEN Cassandra^6^, GARDES Thomas^8^, GINER Franck^1^, GURRIARAN Rodolfo^1^, GRENZ Christian^11^, GROSBOIS Cécile^12^, LESTEL Laurence^13^, LOSSON Benoît^10^, MANSUY-HUAULT Laurence^14^, MONTARGES-PELLETIER Emmanuelle^14^, MOREREAU Amandine^13^, MOURIER Brice^5^, MOURIER David^1^, OLLIVE Vincent^10^, PAPILLON Laure^11^, SCHAFER Jorg^8^, SCHMITT Laurent^6^, SEMPERE Richard^11^, WINIARSKI Thierry^5^, ZEBRACKI Mathilde^15^, EVRARD Olivier^16^

^*^Corresponding author: [frederique.eyrolle@irsn.fr](mailto:frederique.eyrolle@irsn.fr)

**Supplementary Informations**

**SI-1 Geographical context of sediment cores**

***Geographical Context of the Loire Core*** - The 1,012-kilometer-long Loire River begins in the southeastern Massif Central in France, close to Mont Gerbier de Jonc (1,408 m asl). Encompassing a watershed of 117,045 km², it stands as France's largest river basin. The river flows into the Atlantic Ocean near the city of Nantes, with an average annual discharge of 860 m^3^/s. The hydrological patterns of the Loire basin are shaped by a complex interplay of Mediterranean, continental, and Atlantic climates (1). The geological landscape along the course of the Loire River varies, encompassing crystalline and volcanic mountain ranges within the Massif Central, transitioning to sedimentary rocks and alluvial deposits in its middle and lower segment (2). While commonly perceived as a wild river, the Loire has undergone various engineering interventions affecting its hydrosedimentary dynamics. Like many European rivers, dikes were installed in the Middle Ages for flood protection, and groyne fields were introduced to improve navigation conditions in the 19th century. Additionally, considerable sediment extraction took place throughout the latter half of the 20th century. (3, 4). The core has been sampled on an island of the main channel at Montjean-sur-Loire, France, covering 93% (111,172 km²) of the total watershed. Notably, the coring site lies just upstream of a weir, coming from the right bank. Initially spanning the entire secondary channel width, its part close to the island degraded in the early 1980s and potentially modifying hydraulic and sedimentary conditions at the core site.

***Geographical context of the Rhone River core*** *-* The 812-kilometer-long Rhone River originates from the Furka Glacier (2,340 m asl) in Swiss Alps, specifically in the canton of Valais. Spanning a watershed of 98,500 km², it exhibits a complex hydrological regime comprising glacial, nival, and rainfall patterns. The river flows into the Mediterranean Sea, with an average annual discharge of 1,700 m^3^.s^-1^ (5). The Rhône River lithology is complex draining glacial formations as well as sedimentary basin with local occurrences of felsic and mafic rocky outcrops (2). During the latter half of the 20th century, the river underwent significant dam construction, including 17 diversion projects, to meet the demand for hydroelectric power production, ultimately altering the river into a single-thread channel (6, 7, 8). initiated through dike field construction and subsequently reinforced by diversion dam installation, terrestrialization has resulted in the deposition of fine sediment, fluctuations in water levels, and consequent modifications in the hydrological connectivity at the river margins (9). These development history help contextualize the Rhone core, unveiling sediment deposition in the 1960s in an area once submerged. Located upstream of the delta and Arles city, it marks the end of the Lower Rhone section. Covering 98% of the entire watershed, its drained area holds considerable importance.

***Geographical context of the Rhine River core -*** The Rhine River, with a length of 1,233 kilometers, originates in the Swiss Alps at Lake Tomasee. Its extensive watershed spans 185,000 km² across nine European countries. Flowing into the North Sea, its average annual discharge is 2,300 m^3^/s. The Upper Rhine River, which flows from Basel to Bingen-am-Rhein, follows a nivo-glacial hydrological regime, characterized by a mean peak discharge during June-July (10, 11). Before entering France, the Basel gauging station, the river presents an average discharge of 1,050 m3/s (period 1891-2008). The upper Rhine courses through a rift valley filled with deposits ranging from the Oligo-Miocene to the Quaternary period (2). During the mid-19^th^ century, it underwent human interventions, including channelization efforts for flood control, border delineation, agricultural advancement, forest expansion whereas dams were constructed in the 20^th^ century (12, 10, 11, 13). The core site originates from an artificial island formed by redirecting the Rhinau dam, constructed in 1963, within a former braided and anastomosing channel pattern that stabilized due different phases of river regulation works. The basin upstream Rhinau covers approximately 20% of the complete Rhine watershed; it includes, among other countries, parts of Switzerland, Austria, Germany, and France.

***Geographical context of the Seine River core -*** Originating from the Langres Plateau in northeastern France, the 777-kilometer-long Seine River encompasses a watershed of approximately 76,000 km^2^, primarily draining sedimentary calcareous and chalk formations from the Mesozoic and Cenozoic eras. The river concludes its course in the city of Le Havre, flowing into the English Channel, with an average annual discharge of about 500 m3/s. Well-known for its high population density, notably along the Paris-Rouen corridor (4200 inhabitants/km2, cf. (2)), and marked industrialization, especially in metallurgy, the Seine basin has witnessed centuries of significant engineering interventions, including flood protection measures since the Middle Ages and channelization to meet navigation demands (14). The core comes from a connected side channel located in the convexity of the meander of Orival. The drained area associated with the core covers about 94% of the total Seine watershed.

***Geographical context of the Meuse River core -*** The Meuse River, spanning a length of 905 kilometers, originates on the Langres Plateau in the village of Pouilly-en-Bassigny (409 m asl) in France. Its international watershed covers 34,548 km², with significant portions in France, Belgium, and the Netherlands It flows into the North Sea with an average annual discharge of around 320 m^3^/s near the Dutch city of Rotterdam. The prevailing temperate oceanic climate in the basin chiefly determines the hydrological regime of the Meuse, albeit with a discernible continental influence. The Meuse River and its tributaries traverse varied geological formations, encompassing the eastern part of the Paris Basin (Mesozoic calcareous formations), the Ardennes Mountains (metamorphic Palaeozoic rocks), the rolling hills of Belgium (quaternary loamy plateaus), and the lowlands of the Netherlands (peaty-clayey soils) (2). Like many European rivers, the Meuse has experienced significant human impacts, particularly due to its historical importance as a cross-border river with a densely populated watershed (9 million inhabitants). Furthermore, in the highly industrialized area between Liège and Kinrooi, there is a recognized issue of heavy metal contamination in bed sediments (15). While the lower parts of the Meuse are subject to intensive regulation, such as canalization and flood protection, its upper French part is somewhat more preserved, and some sections have retained ecological significance (15, 16). The core site is located in this upper French part, at Givet, just before the Belgian border. It covers a drained area of 10,319 km², which is 31% of the total watershed. On the east bank of the Meuse, the core is situated at the level of a reach bypassed by the Canal de l'Est (Northern Branch), a navigation infrastructure dating from the late 19th century.

***Geographical context of the Moselle River core*** - The 545-kilometer-long Moselle River originates in the Vosges Mountains of northeastern France (715 m asl) at the Bussang Pass. Its watershed of approximately 28,133 km² spans France, Germany, Luxembourg, and Belgium. Converging with the Rhine River at Koblenz, Germany, it exhibits an average annual discharge of 315 m3/s. The French segment of the Moselle flows through diverse geological formations including ancient crystalline rocks like gneiss and granite in the Vosges Massif, varied sedimentary deposits such as limestones, sandstones, and marls in the Lorraine Plateau, and notable geological formations like limestones and marls in the Moselle Valley. The area from Nancy to Thionville is heavily industrialized with steel production, automotive, chemistry, and petrochemical industries (17, 18). A 1956 agreement among France, Germany, and Luxembourg aimed to develop the Moselle into a high-capacity navigable waterway, resulting in channalization efforts between Koblenz and Thionville from 1956 to 1964, facilitating connectivity between Germany and France (19). The core site at Berg-sur-Moselle, France, preceding the Luxembourg border, covers approximately 11,347 km², constituting 40% of the total watershed. A diachronic series in Figure 1.B - *Moselle* displays that the meander at the level of our study site was reshaped between 1955 and 1966, forming an island (*île aux oiseaux de Malling*) between the original meander and the navigation bypass. The core is located within the concavity where hydraulic constraints must have been significant before the modifications but were reduced afterward since the main flow subsequently redirected to the south of the island. The shift from herbaceous to woody vegetation during this period corroborates with decreased hydraulic constraints at the coring site after 1955.

**SI-1 Table 1** Synthesis of analytical data for the Loire, Rhone, Rhine, Seine, Meuse and Moselle sediment cores : Minimum, maximum and mean depth of the sedimentary strata (cm); D10, D50 and D90 (µm) ; Measured ^137^Cs, ^241^Am, ^210^Pb and ^214^Bi concentration (Bq/kg); Apparent Sedimentation Rate (ASR, cm/y); Minimum, maximum and mean age (y); Integrated Period (IP, y); NA: Not Analysed; <LD: Concentration below the limit of detection.

| **River** | **Mean Depth (cm)** | **Min Depth (cm)** | **Max Depth (cm)** | **D10 (µm)** | **D50 (µm)** | **D90 (µm)** | **^137^Cs (Bq/kg)** | **^241^Am (Bq/kg)** | **^210^Pb (Bq/kg)** | **^214^Bi (Bq/kg)** | **ASR (cm/y)** | **Mean age (y)** | **Min age (y)** | **Max age (y)** | **IP (y)** |
| --- | --- | --- | --- | --- | --- | --- | --- | --- | --- | --- | --- | --- | --- | --- | --- |
| LOIRE | 2.5 | 0 | 5 | 2.3 | 15.4 | 73.8 | 7.8 | NA | 125 | 88 | 1.2 | 2018.7 | 2017.3 | 2020.1 | 2.8 |
| LOIRE | 7 | 5 | 9 | 2.5 | 17.8 | 78.8 | 8.7 | 0.131 | 115 | 89 | 1.2 | 2015.0 | 2013.3 | 2016.8 | 3.5 |
| LOIRE | 11 | 9 | 13 | 2.1 | 13.2 | 64.8 | 8.7 | NA | 106 | 90 | 1.2 | 2011.8 | 2010.1 | 2013.4 | 3.3 |
| LOIRE | 15 | 13 | 17 | 2.2 | 14.2 | 69.3 | 8.1 | NA | 107 | 90 | 1.2 | 2008.5 | 2007.0 | 2010.0 | 3.0 |
| LOIRE | 18.5 | 17 | 20 | 2.0 | 12.6 | 61.9 | 8.3 | NA | 95 | 88 | 1.2 | 2005.6 | 2004.3 | 2007.0 | 2.7 |
| LOIRE | 21.5 | 20 | 23 | 2.0 | 13.7 | 77.2 | 8.0 | NA | 91 | 69 | 1.2 | 2003.2 | 2001.9 | 2004.4 | 2.5 |
| LOIRE | 24.5 | 23 | 26 | 2.3 | 15.2 | 73.0 | 8.8 | NA | 82 | 87 | 1.2 | 2000.7 | 1999.5 | 2001.9 | 2.4 |
| LOIRE | 27.5 | 26 | 29 | 2.3 | 15.4 | 78.0 | 9.7 | NA | 86 | 92 | 1.2 | 1998.3 | 1997.0 | 1999.5 | 2.5 |
| LOIRE | 30.5 | 29 | 32 | 2.3 | 17.0 | 85.7 | 12.1 | NA | 86 | 90 | 1.2 | 1995.8 | 1994.6 | 1997 | 2.4 |
| LOIRE | 33.5 | 32 | 35 | 2.2 | 15.6 | 76.5 | 13.3 | NA | 88 | 89 | 1.2 | 1993.4 | 1992.1 | 1994.6 | 2.5 |
| LOIRE | 36.5 | 35 | 38 | 2.0 | 12.8 | 63.1 | 16.2 | NA | 87 | 91 | 1.2 | 1990.9 | 1989.7 | 1992.1 | 2.4 |
| LOIRE | 39.5 | 38 | 41 | 2.0 | 12.9 | 61.3 | 24.0 | NA | 87 | 90 | 1.2 | 1988.5 | 1987.2 | 1989.7 | 2.5 |
| LOIRE | 42.5 | 41 | 44 | 2.4 | 16.2 | 67.2 | 34.9 | NA | 80 | 90 | 1.2 | 1986.0 | 1985.1 | 1986.9 | 1.8 |
| LOIRE | 45.5 | 44 | 47 | 2.3 | 16.0 | 69.2 | 16.7 | 0.120 | 75 | 84 | 3.1 | 1985.0 | 1984.6 | 1985.5 | 0.9 |
| LOIRE | 48.5 | 47 | 50 | 3.0 | 27.3 | 122.9 | 14.0 | 0.135 | 72 | 82 | 3.1 | 1984.1 | 1983.6 | 1984.6 | 1.0 |
| LOIRE | 51.5 | 50 | 53 | 2.3 | 15.6 | 78.9 | 15.0 | 0.21 | 78 | 88 | 3.1 | 1983.1 | 1982.6 | 1983.6 | 1.0 |
| LOIRE | 54.5 | 53 | 56 | 1.9 | 12.4 | 61.9 | 17.2 | 0.263 | 82 | 97 | 3.1 | 1982.2 | 1981.6 | 1982.7 | 1.1 |
| LOIRE | 58 | 56 | 60 | 2.1 | 14.0 | 64.4 | 19.8 | NA | 85 | 98 | 3.1 | 1981.0 | 1980.4 | 1981.6 | 1.2 |
| LOIRE | 62 | 60 | 64 | 2.3 | 17.2 | 78.2 | 16.7 | 0.6 | 83 | 98 | 3.1 | 1979.8 | 1979.1 | 1980.4 | 1.3 |
| LOIRE | 66 | 64 | 68 | 2.2 | 14.6 | 72.3 | 12.9 | 0.295 | 90 | 103 | 3.1 | 1978.5 | 1977.9 | 1979.1 | 1.2 |
| LOIRE | 70 | 68 | 72 | 2.1 | 15.5 | 64.1 | 14.4 | 0.267 | 103 | 118 | 3.1 | 1977.2 | 1976.6 | 1977.9 | 1.3 |
| LOIRE | 74 | 72 | 76 | 2.1 | 14.6 | 62.2 | 15.5 | 0.289 | 102 | 122 | 3.1 | 1975.9 | 1975.3 | 1976.6 | 1.3 |
| LOIRE | 78 | 76 | 80 | 2.1 | 15.1 | 61.2 | 14.4 | 0.359 | 94 | 111 | 3.1 | 1974.7 | 1974.0 | 1975.3 | 1.3 |
| LOIRE | 82 | 80 | 84 | 1.7 | 10.2 | 58.0 | 14.4 | 0.46 | 96 | 115 | 3.1 | 1973.4 | 1972.7 | 1974.0 | 1.3 |
| LOIRE | 86 | 84 | 88 | NA | NA | NA | 15.3 | 0.443 | 89 | 109 | 3.1 | 1972.1 | 1971.5 | 1972.7 | 1.2 |
| LOIRE | 90 | 88 | 92 | 1.8 | 11.1 | 57.3 | 16.6 | 0.415 | 89 | 105 | 3.1 | 1970.8 | 1970.1 | 1971.5 | 1.4 |
| LOIRE | 94.5 | 92 | 97 | 1.9 | 12.9 | 63 | 17.2 | 0.351 | 89 | 98 | 3.1 | 1969.4 | 1968.6 | 1970.1 | 1.5 |
| LOIRE | 99.5 | 97 | 102 | 1.7 | 11.5 | 70.2 | 22.7 | NA | 94 | 98 | 3.1 | 1967.8 | 1967.0 | 1968.6 | 1.6 |
| LOIRE | 104.5 | 102 | 107 | 1.5 | 8.7 | 52.9 | 32.4 | 0.710 | 89 | 97 | 3.1 | 1966.2 | 1965.4 | 1967.0 | 1.6 |
| LOIRE | 109.5 | 107 | 112 | 2.1 | 13.1 | 56.8 | 47.3 | 1.120 | 92 | 99 | 3.1 | 1964.6 | 1963.8 | 1965.4 | 1.6 |
| LOIRE | 114.5 | 112 | 117 | 1.9 | 12.6 | 61.8 | 53.3 | 1.890 | 99 | 104 | 3.1 | 1963.0 | 1962.1 | 1963.9 | 1.8 |
| LOIRE | 119.5 | 117 | 122 | 2.1 | 14.7 | 72.4 | 24.3 | 0.540 | 101 | 104 | 2.5 | 1961.0 | 1960.0 | 1962.0 | 2.0 |
| LOIRE | 124.5 | 122 | 127 | 1.8 | 12.6 | 65.2 | 8.9 | 0.630 | 118 | 109 | 2.5 | 1959.0 | 1958.0 | 1960.0 | 2.0 |
| LOIRE | 129.5 | 127 | 132 | 1.9 | 13.5 | 65.7 | 6.4 | 0.340 | 109 | 97 | 2.5 | 1957.0 | 1956.0 | 1958.0 | 2.0 |
| LOIRE | 134.5 | 132 | 137 | 16.6 | 762.5 | 1369.9 | 0.94 | NA | 67 | 62 | 2.5 | 1955.0 | 1954.1 | 1955.9 | 1.8 |
| LOIRE | 139.5 | 137 | 142 | 369.7 | 651.2 | 1092.7 | <LD | NA | 38 | 37 | 2.8 | 1953.2 | 1952.3 | 1954.1 | 1.8 |
| LOIRE | 144.5 | 142 | 147 | 2.7 | 20.5 | 809.7 | 0.34 | NA | 93 | 88 | 2.8 | 1951.4 | 1950.5 | 1952.3 | 1.8 |
| LOIRE | 149.5 | 147 | 152 | 2.2 | 16.2 | 98.4 | <LD | NA | 76 | 86 | 2.8 | 1949.6 | 1948.7 | 1950.5 | 1.8 |
| LOIRE | 154.5 | 152 | 157 | 2.1 | 15.5 | 79.9 | 0.2 | NA | 88 | 88 | 2.8 | 1947.9 | 1947.0 | 1948.7 | 1.7 |
| LOIRE | 159 | 157 | 161 | 2 | 21.9 | 153.8 | <LD | NA | 74 | 79 | 2.8 | 1946.2 | 1945.5 | 1947.0 | 1.5 |
| LOIRE | 162.5 | 161 | 164 | 4.4 | 180.4 | 507.5 | <LD | NA | 51 | 50 | 2.8 | 1945.0 | 1944.6 | 1945.4 | 0.8 |
| LOIRE | 168 | 164 | 172 | 2.3 | 18.1 | 88.6 | <LD | NA | 88 | 89 | 11.4 | 1944.5 | 1944.2 | 1944.8 | 0.6 |
| LOIRE | 176 | 172 | 180 | 1.9 | 14 | 85.6 | <LD | NA | 83 | 88 | 11.4 | 1943.8 | 1943.5 | 1944.1 | 0.6 |
| LOIRE | 182.5 | 180 | 185 | 2.2 | 17.8 | 95.5 | <LD | NA | 88 | 87 | 11.4 | 1943.2 | 1943.0 | 1943.5 | 0.5 |
| LOIRE | 188 | 185 | 191 | 3.3 | 58.2 | 175.1 | <LD | NA | 76 | 78 | 11.4 | 1942.8 | 1942.5 | 1943.0 | 0.5 |
| LOIRE | 194 | 191 | 197 | 1.8 | 13.9 | 84.5 | <LD | NA | 87 | 87 | 11.4 | 1942.2 | 1941.9 | 1942.5 | 0.6 |
| LOIRE | 202 | 197 | 207 | 2.5 | 32.5 | 164.6 | <LD | NA | 78 | 78 | 11.4 | 1941.5 | 1941.2 | 1941.8 | 0.6 |
| LOIRE | 208 | 207 | 209 | 2.8 | 54.2 | 232.2 | <LD | NA | 68 | 69 | 11.4 | 1941.0 | 1938.7 | 1943.3 | 4.6 |
| LOIRE | 212 | 209 | 215 | 1.9 | 12.9 | 75.0 | <LD | NA | 74 | 77 | 0.5 | 1932.4 | 1927.4 | 1937.5 | 10.1 |
| LOIRE | 217.5 | 215 | 220 | 2.5 | 19.8 | 204.6 | <LD | NA | 73 | 72 | 0.5 | 1920.7 | 1915.1 | 1926.3 | 11.2 |
| LOIRE | 222.5 | 220 | 225 | 93.5 | 543.1 | 1204.2 | <LD | NA | 27 | 26 | 0.5 | 1910.0 | 1910.0 | 1910.0 | 0 |

| **River** | **Mean Depth (cm)** | **Min Depth (cm)** | **Max Depth (cm)** | **D10 (µm)** | **D50 (µm)** | **D90 (µm)** | **^137^Cs (Bq/kg)** | **^241^Am (Bq/kg)** | **^210^Pb (Bq/kg)** | **^214^Bi (Bq/kg)** | **ASR (cm/y)** | **Mean age (y)** | **Min age (y)** | **Max age (y)** | **IP (y)** |
| --- | --- | --- | --- | --- | --- | --- | --- | --- | --- | --- | --- | --- | --- | --- | --- |
| RHONE | 5 | 0 | 10 | 1.4 | 9.5 | 45.1 | 3.22 | NA | 31.1 | 24.2 | 4.39 | 2019.8 | 2018.9 | 2020.6 | 1.7 |
| RHONE | 15 | 10 | 20 | 2.5 | 24.3 | 138.8 | 3.49 | 0.069 | 39.2 | 26.5 | 4.39 | 2017.5 | 2016.4 | 2018.6 | 2.2 |
| RHONE | 25 | 20 | 30 | 2.2 | 18.2 | 92.1 | 4.38 | NA | 42.5 | 29.4 | 4.39 | 2015.2 | 2014.1 | 2016.4 | 2.3 |
| RHONE | 35 | 30 | 40 | 2.8 | 20.8 | 82.4 | 5.9 | NA | 47.7 | 33.1 | 4.39 | 2012.9 | 2011.8 | 2014.1 | 2.3 |
| RHONE | 45.5 | 40 | 51 | 2.6 | 20.4 | 80.7 | 7.7 | 0.158 | 41.4 | 33 | 4.39 | 2010.5 | 2009.5 | 2011.6 | 2.1 |
| RHONE | 53.5 | 51 | 56 | 3.2 | 23.5 | 86.5 | 5.5 | NA | 41.3 | 37 | 4.39 | 2008.7 | 2008.1 | 2009.4 | 1.3 |
| RHONE | 57 | 56 | 58 | 3.5 | 31.7 | 110.2 | 5.1 | NA | 39.6 | 37.3 | 4.39 | 2007.9 | 2007.4 | 2008.5 | 1.1 |
| RHONE | 63 | 58 | 68 | 1.9 | 17.0 | 78.4 | 5.2 | NA | 27.8 | 24.1 | 4.39 | 2006.6 | 2005.8 | 2007.3 | 1.5 |
| RHONE | 70.75 | 68 | 73.5 | 2.1 | 19.9 | 82.3 | 4.68 | 0.076 | 27.7 | 23.1 | 4.39 | 2004.8 | 2003.9 | 2005.7 | 1.8 |
| RHONE | 79.25 | 73.5 | 85 | 1.7 | 12.1 | 60.5 | 8.4 | NA | 39.4 | 30.3 | 4.39 | 2002.8 | 2001.8 | 2003.9 | 2.1 |
| RHONE | 90 | 85 | 95 | 1.6 | 12.1 | 54.0 | 9.6 | NA | 34 | 28.9 | 4.39 | 2000.4 | 1999.3 | 2001.5 | 2.2 |
| RHONE | 97.75 | 95 | 100.5 | 1.8 | 14.9 | 65.9 | 11.1 | 0.2 | 32.2 | 28.3 | 4.39 | 1998.6 | 1997.8 | 1999.4 | 1.6 |
| RHONE | 103.75 | 100.5 | 107 | 1.8 | 12.6 | 51.9 | 16.5 | 0.509 | 43.6 | 36.9 | 4.39 | 1997.3 | 1996.6 | 1997.9 | 1.3 |
| RHONE | 109.5 | 107 | 112 | 1.9 | 13.3 | 51.5 | 16.9 | 0.41 | 40.3 | 37.9 | 4.39 | 1995.9 | 1995.3 | 1996.6 | 1.3 |
| RHONE | 115 | 112 | 118 | 2.5 | 15.0 | 49.3 | 18.7 | 0.508 | 52 | 47 | 4.39 | 1994.7 | 1994.0 | 1995.4 | 1.4 |
| RHONE | 121.5 | 118 | 125 | 2.2 | 14.0 | 49.5 | 27.4 | 0.64 | 55 | 48 | 4.39 | 1993.2 | 1992.5 | 1994.0 | 1.5 |
| RHONE | 128 | 125 | 131 | 2.3 | 14.4 | 49.9 | 22.2 | 0.736 | 57 | 57 | 4.39 | 1991.7 | 1990.9 | 1992.5 | 1.6 |
| RHONE | 135.5 | 131 | 140 | 2.5 | 17.4 | 58.9 | 73.0 | 2.92 | 57 | 48 | 4.39 | 1990.0 | 1988.9 | 1991.2 | 2.3 |
| RHONE | 145.5 | 140 | 151 | 3.3 | 28.1 | 87.2 | 60.0 | 3.07 | 46.8 | 44.2 | 3.56 | 1987.2 | 1985.8 | 1988.6 | 2.8 |
| RHONE | 155.5 | 151 | 160 | 2.9 | 22.8 | 80.7 | 24.2 | 1.17 | 53 | 52 | 3.56 | 1984.4 | 1983.2 | 1985.6 | 2.4 |
| RHONE | 162 | 160 | 164 | 3.4 | 23.7 | 81 | 20.1 | 1.01 | 44.8 | 44.9 | 3.56 | 1982.6 | 1981.8 | 1983.3 | 1.5 |
| RHONE | 166 | 164 | 168 | 2.4 | 16.9 | 68.5 | 17.0 | 0.43 | 40.1 | 41.8 | 3.56 | 1981.4 | 1980.8 | 1982.1 | 1.3 |
| RHONE | 171 | 168 | 174 | 2.4 | 19.4 | 75.9 | 41.6 | 1.82 | 44.3 | 44.5 | 3.56 | 1980.0 | 1979.2 | 1980.8 | 1.6 |
| RHONE | 177.5 | 174 | 181 | 3.0 | 22.0 | 75.3 | 58.0 | 2.9 | 44.3 | 43 | 3.56 | 1978.2 | 1977.2 | 1979.2 | 2.0 |
| RHONE | 184.75 | 181 | 188.5 | 2.7 | 22.3 | 78.0 | 19.0 | 0.782 | 44.5 | 43.1 | 3.56 | 1976.2 | 1975.1 | 1977.3 | 2.2 |
| RHONE | 193.25 | 188.5 | 198 | 3.3 | 24.7 | 95.0 | 28.9 | 1.24 | 45.3 | 45.2 | 3.56 | 1973.8 | 1972.6 | 1974.9 | 2.3 |
| RHONE | 201 | 198 | 204 | 4.5 | 32.3 | 96.1 | 30.0 | 0.743 | 49.5 | 48 | 3.56 | 1971.6 | 1970.6 | 1972.5 | 1.9 |
| RHONE | 206.75 | 204 | 209.5 | 2.8 | 23.5 | 88.2 | 31.8 | 1.42 | 35.7 | 37.2 | 3.56 | 1970.0 | 1969.0 | 1970.9 | 1.9 |
| RHONE | 214.75 | 209.5 | 220 | 2.8 | 26.6 | 120.2 | 19.3 | 0.449 | 36.1 | 36.8 | 3.56 | 1967.7 | 1966.6 | 1968.9 | 2.3 |
| RHONE | 223.5 | 220 | 227 | 3.3 | 21.6 | 71.6 | 13.2 | 0.132 | 36.4 | 39.1 | 3.56 | 1965.3 | 1964.1 | 1966.4 | 2.3 |
| RHONE | 231.5 | 227 | 236 | 3.3 | 20.5 | 76.4 | 47.3 | NA | 38.5 | 37 | 3.56 | 1963 | 1961.7 | 1964.3 | 2.6 |
| RHONE | 238.5 | 236 | 241 | 4.8 | 46.1 | 158.9 | 9.5 | 0.149 | 30.4 | 30.4 | 2.38 | 1960.1 | 1958.7 | 1961.4 | 2.7 |
| RHONE | 244.5 | 241 | 248 | 2.5 | 25.6 | 115.7 | 2.96 | NA | 27.4 | 28.6 | 2.38 | 1957.5 | 1956.3 | 1958.8 | 2.5 |
| RHONE | 250.5 | 248 | 253 | 3.9 | 57.7 | 189.2 | 0.68 | 0.033 | 23.7 | 24 | 2.38 | 1955.0 | 1953.7 | 1956.3 | 2.6 |
| RHONE | 257 | 253 | 261 | 12.7 | 98 | 307.9 | 0.36 | NA | 24.4 | 26.4 | 2.38 | 1952.3 | 1950.8 | 1953.8 | 3.0 |
| RHONE | 265 | 261 | 269 | 5.3 | 41.7 | 169.0 | 0.51 | NA | 28.8 | 28.1 | 2.38 | 1948.9 | 1947.1 | 1950.8 | 3.7 |
| RHONE | 274.5 | 269 | 280 | 2.1 | 19.4 | 100.8 | 2.02 | NA | 23.9 | 24.8 | 2.38 | 1944.9 | 1942.6 | 1947.2 | 4.6 |
| RHONE | 287 | 280 | 294 | 4.3 | 36 | 105.8 | 0.79 | NA | 27.2 | 28.2 | 2.38 | 1939.7 | 1936.8 | 1942.5 | 5.7 |
| RHONE | 302 | 294 | 310 | 2.0 | 15.4 | 76.3 | 0.82 | 0.023 | 31.2 | 32.7 | 2.38 | 1933.3 | 1930.3 | 1936.3 | 6.0 |
| RHONE | 315.5 | 310 | 321 | 2.4 | 24.2 | 119.0 | 0.48 | NA | 28.9 | 27.3 | 2.38 | 1927.7 | 1925.2 | 1930.1 | 4.9 |
| RHONE | 325.5 | 321 | 330 | 1.8 | 15.1 | 78.8 | 0.4 | NA | 24.6 | 25.9 | 2.38 | 1923.4 | 1921.7 | 1925.2 | 3.5 |
| RHONE | 332 | 330 | 334 | 2.2 | 16.4 | 107.5 | 0.48 | NA | 32.8 | 32.7 | 2.38 | 1920.7 | 1919.4 | 1922.0 | 2.6 |
| RHONE | 338 | 334 | 342 | 4.6 | 44.5 | 161.4 | <LD | 0.003 | 27.6 | 28.1 | 2.38 | 1918.2 | 1916.0 | 1919.5 | 3.5 |

| **River** | **Mean Depth (cm)** | **Min Depth (cm)** | **Max Depth (cm)** | **D10 (µm)** | **D50 (µm)** | **D90 (µm)** | **^137^Cs (Bq/kg)** | **^241^Am (Bq/kg)** | **^210^Pb (Bq/kg)** | **^214^Bi (Bq/kg)** | **ASR (cm/y)** | **Mean age (y)** | **Min age (y)** | **Max age (y)** | **IP (y)** |
| --- | --- | --- | --- | --- | --- | --- | --- | --- | --- | --- | --- | --- | --- | --- | --- |
| RHINE | 0.5 | 0 | 1 | 4.5 | 25.3 | 202.0 | 10 | NA | 86 | 31.9 | 0.6 | 2020.4 | 2019.8 | 2021.0 | 1.2 |
| RHINE | 1.5 | 1 | 2 | 3.7 | 29.8 | 121.1 | 11.6 | NA | 86 | 31.2 | 0.6 | 2018.7 | 2017.9 | 2019.6 | 1.7 |
| RHINE | 2.5 | 2 | 3 | 3.5 | 30.7 | 113.9 | 11.7 | NA | 83 | 30.4 | 0.6 | 2017.1 | 2016.2 | 2017.9 | 1.7 |
| RHINE | 3.5 | 3 | 4 | 2.9 | 24.8 | 107.9 | 14.1 | 0.079 | 76 | 31.4 | 0.6 | 2015.4 | 2014.5 | 2016.2 | 1.7 |
| RHINE | 4.5 | 4 | 5 | 3.0 | 30.2 | 118.4 | 19.1 | NA | 74 | 33 | 0.6 | 2013.7 | 2012.9 | 2014.5 | 1.6 |
| RHINE | 5.5 | 5 | 6 | 3.1 | 16.0 | 56.8 | 20.7 | NA | 75 | 32.5 | 0.6 | 2012.0 | 2011.2 | 2012.9 | 1.7 |
| RHINE | 6.5 | 6 | 7 | 2.7 | 27.1 | 110.9 | 19.3 | NA | 72 | 31.9 | 0.6 | 2010.3 | 2009.5 | 2011.2 | 1.7 |
| RHINE | 7.5 | 7 | 8 | 2.8 | 30.2 | 116.8 | 25.1 | NA | 76 | 32 | 0.6 | 2008.7 | 2007.8 | 2009.5 | 1.7 |
| RHINE | 8.5 | 8 | 9 | 2.7 | 72.8 | 640.3 | 30 | NA | 69 | 32 | 0.6 | 2007.0 | 2006.1 | 2007.8 | 1.7 |
| RHINE | 9.5 | 9 | 10 | 2.4 | 12.3 | 42.9 | 39 | NA | 72 | 33.4 | 0.6 | 2005.3 | 2004.3 | 2006.4 | 2.1 |
| RHINE | 11 | 10 | 12 | 2.3 | 12.6 | 41.5 | 41.4 | NA | 74 | 35.7 | 0.6 | 2002.8 | 2001.3 | 2004.3 | 3 |
| RHINE | 13 | 12 | 14 | 2.2 | 23.5 | 106.9 | 47.4 | NA | 66 | 34.9 | 0.6 | 1999.4 | 1997.8 | 2001.1 | 3.3 |
| RHINE | 15 | 14 | 16 | 2.1 | 21.8 | 105.3 | 52.7 | 0.141 | 61 | 33.5 | 0.6 | 1996.1 | 1994.4 | 1997.8 | 3.4 |
| RHINE | 17 | 16 | 18 | 2.1 | 20.9 | 106.9 | 66 | NA | 60 | 34.2 | 0.6 | 1992.7 | 1991 | 1994.4 | 3.4 |
| RHINE | 19 | 18 | 20 | 2.1 | 23.7 | 109.3 | 81 | NA | 64 | 36 | 0.6 | 1989.4 | 1987.7 | 1991.0 | 3.3 |
| RHINE | 21 | 20 | 22 | 1.9 | 14.7 | 96.6 | 91 | 0.221 | 53 | 37.1 | 0.6 | 1986.0 | 1983.2 | 1988.8 | 5.6 |
| RHINE | 23 | 22 | 24 | 1.8 | 23.5 | 113.5 | 65 | NA | 48 | 35.9 | 0.26 | 1978.3 | 1974.5 | 1982.2 | 7.7 |
| RHINE | 25 | 24 | 26 | 1.8 | 22.4 | 106.7 | 50.2 | NA | 41.3 | 34 | 0.26 | 1970.7 | 1966.8 | 1974.5 | 7.7 |
| RHINE | 27 | 26 | 28 | 1.9 | 21.2 | 102.1 | 36.2 | 0.272 | 42.8 | 33 | 0.26 | 1963.0 | 1960.8 | 1965.2 | 4.4 |
| RHINE | 29 | 28 | 30 | 2.0 | 26.8 | 112.3 | 31.1 | NA | 39.7 | 32.4 | 1.75 | 1961.9 | 1961.2 | 1962.5 | 1.3 |
| RHINE | 31.75 | 30 | 33.5 | 2.4 | 27.1 | 106.9 | 19.3 | NA | 35.5 | 31.5 | 1.75 | 1960.3 | 1959.5 | 1961.1 | 1.6 |
| RHINE | 34.75 | 33.5 | 36 | 1.2 | 9.6 | 42.6 | 13 | 0.143 | 35.6 | 30.9 | 1.75 | 1958.6 | 1957.8 | 1959.3 | 1.5 |
| RHINE | 37 | 36 | 38 | 1.7 | 33.4 | 121.5 | 6.6 | NA | 29.2 | 30.3 | 1.75 | 1957.3 | 1956.7 | 1957.9 | 1.2 |
| RHINE | 39 | 38 | 40 | 1.5 | 10.6 | 48.5 | 3.11 | NA | 26.6 | 28.1 | 1.75 | 1956.1 | 1955.6 | 1956.7 | 1.1 |
| RHINE | 41 | 40 | 42 | 1.5 | 27.8 | 110.9 | 1.98 | 0.027 | 29.7 | 28.1 | 1.75 | 1955.0 | 1953.8 | 1956.2 | 2.4 |
| RHINE | 43 | 42 | 44 | 1.5 | 29.9 | 115.9 | 1.94 | NA | 26.7 | 30.5 | 0.53 | 1951.3 | 1949.4 | 1953.1 | 3.7 |
| RHINE | 45 | 44 | 46 | 1.5 | 30.0 | 110.9 | 0.85 | NA | 27.8 | 29.8 | 0.53 | 1947.5 | 1945.6 | 1949.4 | 3.8 |
| RHINE | 47 | 46 | 48 | 1.4 | 26.3 | 116.4 | 0.65 | 0.0096 | 27.5 | 29.7 | 0.53 | 1943.8 | 1941.9 | 1945.6 | 3.7 |
| RHINE | 49 | 48 | 50 | 1.6 | 9.3 | 44.0 | 0.66 | NA | 29.3 | 29.4 | 0.53 | 1940.0 | 1938.2 | 1941.9 | 3.7 |
| RHINE | 51 | 50 | 52 | 1.4 | 27.1 | 108.9 | 0.84 | NA | 29.8 | 30.1 | 0.53 | 1936.3 | 1934.2 | 1938.4 | 4.2 |
| RHINE | 53.5 | 52 | 55 | 1.2 | 9.3 | 43.7 | 1.85 | 0.0092 | 28.5 | 30.3 | 0.53 | 1931.6 | 1929.0 | 1934.2 | 5.2 |
| RHINE | 56.5 | 55 | 58 | 1.2 | 27.4 | 112.3 | 1.82 | NA | 31.7 | 30.7 | 0.53 | 1926.0 | 1923.2 | 1928.8 | 5.6 |
| RHINE | 59.5 | 58 | 61 | 1.5 | 24.8 | 107.6 | 0.95 | NA | 28.2 | 30 | 0.53 | 1920.4 | 1917.3 | 1923.4 | 6.1 |
| RHINE | 63 | 61 | 65 | 1.2 | 22.8 | 110.2 | 0.81 | 0.0058 | 29.9 | 30.5 | 0.53 | 1913.8 | 1911.0 | 1916.6 | 5.6 |
| RHINE | 65.5 | 65 | 66 | 1.2 | 23.3 | 121.6 | 0.83 | NA | 32.7 | 31.7 | 0.53 | 1909.1 | 1906.8 | 1911.5 | 4.7 |
| RHINE | 68 | 66 | 70 | 1.5 | 10.3 | 74.9 | 1.01 | 0.0056 | 31.2 | 29.7 | 0.53 | 1904.5 | 1901.7 | 1907.3 | 5.6 |
| RHINE | 71.5 | 70 | 73 | 2.2 | 44.5 | 139.4 | 1.34 | NA | 22.3 | 17.9 | 0.53 | 1897.9 | 1895.0 | 1900.8 | 5.8 |
| RHINE | 74.25 | 73 | 75.5 | 2.6 | 49.6 | 137.1 | 0.33 | NA | 21.6 | 18.8 | 0.53 | 1892.8 | 1889.3 | 1896.3 | 7 |
| RHINE | 79 | 75.5 | 82.5 | 2.3 | 39.6 | 125.1 | 0.18 | NA | 24 | 23 | 0.53 | 1883.9 | 1879.4 | 1888.3 | 8.9 |

| **River** | **Mean Depth (cm)** | **Min Depth (cm)** | **Max Depth (cm)** | **D10 (µm)** | **D50 (µm)** | **D90 (µm)** | **^137^Cs (Bq/kg)** | **^241^Am (Bq/kg)** | **^210^Pb (Bq/kg)** | **^214^Bi (Bq/kg)** | **ASR (cm/y)** | **Mean age (y)** | **Min age (y)** | **Max age (y)** | **IP (y)** |
| --- | --- | --- | --- | --- | --- | --- | --- | --- | --- | --- | --- | --- | --- | --- | --- |
| SEINE | 2.25 | 0 | 4.5 | 10.3 | 83.6 | 209.6 | 1.03 | NA | 32.3 | 16.9 | 5.1 | 2021.5 | 2021.3 | 2021.7 | 0.4 |
| SEINE | 5.25 | 4.5 | 6 | 4.0 | 35.4 | 153.0 | 2.78 | NA | 51 | 24.8 | 5.1 | 2020.9 | 2020.4 | 2021.4 | 1.0 |
| SEINE | 12.5 | 6 | 19 | 6.6 | 93.5 | 233.3 | 0.65 | 0.16 | 16.4 | 12.6 | 5.1 | 2019.5 | 2018.5 | 2020.5 | 2.0 |
| SEINE | 25.5 | 19 | 32 | 47.9 | 153.9 | 274.7 | 0.61 | NA | 17.9 | 12.4 | 5.1 | 2017.0 | 2015.8 | 2018.2 | 2.4 |
| SEINE | 37 | 32 | 42 | 7.3 | 77.6 | 189.5 | 1.11 | NA | 27.2 | 15.6 | 5.1 | 2014.7 | 2013.6 | 2015.8 | 2.2 |
| SEINE | 48.5 | 42 | 55 | 5.1 | 42.3 | 151.8 | 1.36 | NA | 31 | 18.6 | 5.1 | 2012.5 | 2011.4 | 2013.6 | 2.2 |
| SEINE | 59.5 | 55 | 64 | 4.2 | 36.1 | 139 | 1.26 | NA | 25.2 | 18 | 5.1 | 2010.4 | 2009.4 | 2011.3 | 1.9 |
| SEINE | 68.5 | 64 | 73 | 4.1 | 29.6 | 98.7 | 1.88 | 0.062 | 35.1 | 21.7 | 5.1 | 2008.6 | 2007.7 | 2009.5 | 1.8 |
| SEINE | 78.5 | 73 | 84 | 3.8 | 27.5 | 89.8 | 2.1 | NA | 38.7 | 25.1 | 5.1 | 2006.7 | 2005.6 | 2007.7 | 2.1 |
| SEINE | 89.25 | 84 | 94.5 | 4.1 | 31.0 | 120.1 | 1.54 | NA | 31.7 | 20.6 | 5.1 | 2004.6 | 2003.6 | 2005.5 | 1.9 |
| SEINE | 97.75 | 94.5 | 101 | 2.7 | 19.7 | 63.8 | 3.57 | NA | 51 | 34.5 | 5.1 | 2002.9 | 2002.2 | 2003.6 | 1.4 |
| SEINE | 103 | 101 | 105 | 2.9 | 20.1 | 62.7 | 1.83 | NA | 33.5 | 22.2 | 5.1 | 2001.9 | 2001.4 | 2002.3 | 0.9 |
| SEINE | 107 | 105 | 109 | 3.5 | 23.4 | 69.9 | 2.71 | NA | 44 | 28.6 | 5.1 | 2001.1 | 2000.6 | 2001.6 | 1.0 |
| SEINE | 113.5 | 109 | 118 | 3.7 | 27.1 | 88.2 | 3.89 | NA | 52 | 32.1 | 5.1 | 1999.9 | 1998.9 | 2000.8 | 1.9 |
| SEINE | 126.25 | 118 | 134.5 | 3.0 | 20.4 | 63.9 | 4.53 | NA | 51 | 35.1 | 5.1 | 1997.4 | 1996.2 | 1998.6 | 2.4 |
| SEINE | 137.75 | 134.5 | 141 | 5.5 | 35.7 | 120.8 | 4.46 | NA | 40.3 | 30.5 | 5.1 | 1995.1 | 1994.3 | 1995.9 | 1.6 |
| SEINE | 142.5 | 141 | 144 | 3.9 | 33.4 | 114.3 | 2.62 | NA | 34.6 | 24.1 | 5.1 | 1994.2 | 1993.8 | 1994.6 | 0.8 |
| SEINE | 145.5 | 144 | 147 | 4.4 | 30.7 | 109.6 | 3.87 | NA | 39.6 | 28.3 | 5.1 | 1993.6 | 1993.1 | 1994.1 | 1 |
| SEINE | 153 | 147 | 159 | 2.3 | 16.3 | 52.7 | 5.9 | NA | 55 | 37.9 | 5.1 | 1992.2 | 1991.4 | 1993.0 | 1.6 |
| SEINE | 162 | 159 | 165 | 4.1 | 28.9 | 96.9 | 4.45 | NA | 42.4 | 30.8 | 5.1 | 1990.4 | 1989.7 | 1991.2 | 1.5 |
| SEINE | 168.25 | 165 | 171.5 | 3.1 | 22.1 | 64.4 | 7.0 | 0.096 | 51 | 37.3 | 5.1 | 1989.2 | 1988.5 | 1989.9 | 1.4 |
| SEINE | 176.5 | 171.5 | 181.5 | 4.7 | 29.6 | 151.4 | 7.8 | 0.39 | 45.3 | 34.3 | 5.1 | 1987.6 | 1986.8 | 1988.4 | 1.6 |
| SEINE | 184.75 | 181.5 | 188 | 3.5 | 25.0 | 82.3 | 14.9 | 0.377 | 47 | 39.8 | 5.1 | 1986.0 | 1985.3 | 1986.7 | 1.4 |
| SEINE | 193.5 | 188 | 199 | 3.8 | 25.5 | 80.5 | 11.8 | NA | 45 | 35.5 | 7.1 | 1984.8 | 1984.1 | 1985.4 | 1.3 |
| SEINE | 203 | 199 | 207 | 4.3 | 31.1 | 107.2 | 8.9 | 0.238 | 43 | 33.8 | 7.1 | 1983.4 | 1982.8 | 1984.0 | 1.2 |
| SEINE | 211 | 207 | 215 | 5.1 | 33.7 | 104.4 | 6.2 | 0.34 | 31 | 26.9 | 7.1 | 1982.3 | 1981.8 | 1982.9 | 1.1 |
| SEINE | 218.5 | 215 | 222 | 4.6 | 34.7 | 139.5 | 3.97 | 0.103 | 24.9 | 19.7 | 7.1 | 1981.3 | 1980.8 | 1981.7 | 0.9 |
| SEINE | 225 | 222 | 228 | 5.8 | 86.1 | 305.9 | 1.21 | NA | 15.1 | 12.1 | 7.1 | 1980.3 | 1979.9 | 1980.8 | 0.9 |
| SEINE | 231.5 | 228 | 235 | 4.6 | 30.7 | 111.5 | 3.65 | 0.107 | 24.1 | 21.6 | 7.1 | 1979.4 | 1979 | 1979.9 | 0.9 |
| SEINE | 238 | 235 | 241 | 4.4 | 40.6 | 248.6 | 2.34 | NA | 21.4 | 18.8 | 7.1 | 1978.5 | 1978.0 | 1979.0 | 1.0 |
| SEINE | 245 | 241 | 249 | 3.1 | 34.4 | 288.8 | 5.39 | NA | 23.5 | 21.4 | 7.1 | 1977.5 | 1977.0 | 1978.0 | 1.0 |
| SEINE | 252 | 249 | 255 | 2.5 | 16.3 | 83.7 | 1.13 | NA | 35.8 | 31.8 | 7.1 | 1976.5 | 1976.1 | 1977.0 | 0.9 |
| SEINE | 258.5 | 255 | 262 | 2.6 | 17.1 | 86.5 | 0.81 | NA | 40.8 | 34.5 | 7.1 | 1975.6 | 1975.1 | 1976.2 | 1.1 |
| SEINE | 268 | 262 | 274 | 2.5 | 16.1 | 108.4 | 8.9 | NA | 35.4 | 29.1 | 7.1 | 1974.3 | 1973.5 | 1975.0 | 1.5 |
| SEINE | 280 | 274 | 286 | 2.0 | 17.7 | 158.4 | 2.97 | NA | 19.4 | 17.2 | 7.1 | 1972.6 | 1971.8 | 1973.4 | 1.6 |
| SEINE | 291.5 | 286 | 297 | 2.5 | 22.6 | 169.9 | 1.45 | NA | 26.2 | 24 | 7.1 | 1971.0 | 1970.2 | 1971.7 | 1.5 |
| SEINE | 301 | 297 | 305 | 2.1 | 21.6 | 235.2 | 1.4 | NA | 19.8 | 19.3 | 7.1 | 1969.6 | 1969.0 | 1970.2 | 1.2 |
| SEINE | 308.5 | 305 | 312 | 2.8 | 86.7 | 405.6 | 1.42 | NA | 20.1 | 16.5 | 7.1 | 1968.6 | 1968.1 | 1969.1 | 1.0 |
| SEINE | 315.5 | 312 | 319 | 2.7 | 23.7 | 178.8 | 3.35 | NA | 24.4 | 21.1 | 7.1 | 1967.6 | 1967.0 | 1968.2 | 1.2 |
| SEINE | 324.5 | 319 | 330 | 2.3 | 19.4 | 147.4 | 3.35 | NA | 25.7 | 20.9 | 7.1 | 1966.3 | 1965.6 | 1967.1 | 1.5 |
| SEINE | 337.25 | 330 | 344.5 | 2.2 | 15.1 | 88.5 | 8.8 | NA | 35.7 | 29.3 | 7.1 | 1964.5 | 1963.7 | 1965.4 | 1.7 |
| SEINE | 348.25 | 344.5 | 352 | 2.9 | 25.9 | 123.6 | 11.8 | 0.42 | 30.3 | 28.5 | 7.1 | 1963.0 | 1962.4 | 1963.6 | 1.2 |
| SEINE | 356 | 352 | 360 | 2.4 | 22.0 | 145.1 | 10.4 | NA | 32.6 | 26.5 | 8.4 | 1962.1 | 1961.6 | 1962.5 | 0.9 |
| SEINE | 363.5 | 360 | 367 | 3.0 | 24.4 | 166.9 | 7.0 | NA | 34.6 | 30.2 | 8.4 | 1961.2 | 1960.8 | 1961.6 | 0.8 |
| SEINE | 370.5 | 367 | 374 | 2.0 | 18.9 | 115.1 | 1.22 | NA | 22.2 | 21.2 | 8.4 | 1960.4 | 1959.9 | 1960.8 | 0.9 |
| SEINE | 379 | 374 | 384 | 74.7 | 221.8 | 462.7 | 0.86 | NA | 12.2 | 11.4 | 8.4 | 1959.3 | 1958.8 | 1959.9 | 1.1 |
| SEINE | 389 | 384 | 394 | 2.5 | 45.1 | 319.5 | 1.24 | NA | 13.7 | 13.1 | 8.4 | 1958.2 | 1957.6 | 1958.7 | 1.1 |
| SEINE | 398.5 | 394 | 403 | 3.1 | 88.5 | 437.2 | 1.31 | NA | 13.5 | 13.9 | 8.4 | 1957.0 | 1956.5 | 1957.6 | 1.1 |
| SEINE | 407.5 | 403 | 412 | 1.9 | 12.0 | 85.8 | 0.45 | NA | 21.7 | 19.9 | 8.4 | 1956.0 | 1955.4 | 1956.5 | 1.1 |
| SEINE | 415.5 | 412 | 419 | 2.2 | 18.8 | 127.7 | <LD | NA | 22.5 | 21.3 | 8.4 | 1955.0 | 1954.5 | 1955.5 | 1.0 |

| **River** | **Mean Depth (cm)** | **Min Depth (cm)** | **Max Depth (cm)** | **D10 (µm)** | **D50 (µm)** | **D90 (µm)** | **^137^Cs (Bq/kg)** | **^241^Am (Bq/kg)** | **^210^Pb (Bq/kg)** | **^214^Bi (Bq/kg)** | **ASR (cm/y)** | **Mean age (y)** | **Min age (y)** | **Max age (y)** | **IP (y)** |
| --- | --- | --- | --- | --- | --- | --- | --- | --- | --- | --- | --- | --- | --- | --- | --- |
| MEUSE | 1 | 0 | 2 | 3.1 | 18.4 | 84.4 | 8.9 | NA | 83 | 41.8 | 0.5 | 2021.2 | 2019.1 | 2022.0 | 2.9 |
| MEUSE | 3 | 2 | 4 | 2.8 | 16.2 | 74.7 | 8.5 | NA | 77 | 41.8 | 0.5 | 2016.7 | 2014.8 | 2018.7 | 3.9 |
| MEUSE | 5 | 4 | 6 | 2.9 | 17.6 | 77.7 | 8.8 | NA | 70 | 42.4 | 0.5 | 2012.9 | 2011 | 2014.8 | 3.8 |
| MEUSE | 7 | 6 | 8 | 2.8 | 16.7 | 74.5 | 8.9 | NA | 62 | 40.8 | 0.5 | 2009.1 | 2007.1 | 2011.0 | 3.9 |
| MEUSE | 9 | 8 | 10 | 2.9 | 17.7 | 90.1 | 9.2 | NA | 61 | 42.8 | 0.5 | 2005.2 | 2003.3 | 2007.1 | 3.8 |
| MEUSE | 11 | 10 | 12 | 2.8 | 17.5 | 77.9 | 10.2 | NA | 64 | 40.7 | 0.5 | 2001.4 | 1999.4 | 2003.3 | 3.9 |
| MEUSE | 13 | 12 | 14 | 2.7 | 17.2 | 81 | 11.7 | NA | 57 | 42.7 | 0.5 | 1997.5 | 1995.6 | 1999.4 | 3.8 |
| MEUSE | 15 | 14 | 16 | 2.7 | 16.3 | 77.4 | 12.9 | NA | 55 | 41.2 | 0.5 | 1993.7 | 1991.8 | 1995.6 | 3.8 |
| MEUSE | 17 | 16 | 18 | 2.6 | 15.9 | 77.2 | 13.7 | NA | 53 | 40.7 | 0.5 | 1989.8 | 1987.9 | 1991.8 | 3.9 |
| MEUSE | 19 | 18 | 20 | 2.6 | 15.7 | 77.9 | 15.9 | NA | 52 | 40.3 | 0.5 | 1986.0 | 1984.3 | 1987.7 | 3.4 |
| MEUSE | 21 | 20 | 22 | 2.5 | 15.0 | 74.8 | 13.6 | NA | 46 | 35.7 | 0.7 | 1983.0 | 1981.5 | 1984.5 | 3.0 |
| MEUSE | 23 | 22 | 24 | 2.5 | 15.3 | 75.8 | 15.4 | NA | 49 | 41.1 | 0.7 | 1980.0 | 1978.5 | 1981.5 | 3.0 |
| MEUSE | 25 | 24 | 26 | 2.5 | 15.4 | 75.6 | 17.0 | NA | 53 | 43.7 | 0.7 | 1977.0 | 1975.5 | 1978.5 | 3.0 |
| MEUSE | 27 | 26 | 28 | 2.5 | 15.5 | 76.5 | 18.8 | NA | 47 | 43.8 | 0.7 | 1974.0 | 1972.5 | 1975.5 | 3.0 |
| MEUSE | 29 | 28 | 30 | 2.5 | 16.0 | 74.4 | 21.3 | NA | 47 | 42.8 | 0.7 | 1971.0 | 1969.9 | 1972.1 | 2.2 |
| MEUSE | 31 | 30 | 32 | 2.5 | 15.9 | 77.9 | 20.7 | NA | 50 | 42.1 | 1.6 | 1969.8 | 1969.2 | 1970.4 | 1.2 |
| MEUSE | 33 | 32 | 34 | 2.5 | 15.7 | 72.1 | 18.5 | NA | 48 | 42.6 | 1.6 | 1968.5 | 1968.0 | 1969.1 | 1.1 |
| MEUSE | 34.75 | 34 | 35.5 | 2.6 | 17.5 | 87.7 | 18.2 | NA | 41 | 28.2 | 1.6 | 1967.5 | 1967.0 | 1968.0 | 1.0 |
| MEUSE | 36.25 | 35.5 | 37 | 2.8 | 20.2 | 120.2 | 16.3 | NA | 42 | 35.4 | 1.6 | 1966.5 | 1966.0 | 1967.0 | 1.0 |
| MEUSE | 38 | 37 | 39 | 2.6 | 17.5 | 86.7 | 21.4 | 0.74 | 45 | 37.9 | 1.6 | 1965.5 | 1964.9 | 1966.0 | 1.1 |
| MEUSE | 40 | 39 | 41 | 2.5 | 15.8 | 73.4 | 26.4 | 0.85 | 44 | 39.6 | 1.6 | 1964.2 | 1963.6 | 1964.8 | 1.2 |
| MEUSE | 42 | 41 | 43 | 2.5 | 15.6 | 76.4 | 28.5 | 0.66 | 43 | 41.1 | 1.6 | 1963.0 | 1962.5 | 1963.5 | 1.0 |
| MEUSE | 44 | 43 | 45 | 2.6 | 15.6 | 70.4 | 26.0 | 0.66 | 43.0 | 41.3 | 2.5 | 1962.2 | 1961.8 | 1962.6 | 0.8 |
| MEUSE | 46 | 45 | 47 | 2.6 | 16 | 72.2 | 21.5 | NA | 52.0 | 42.8 | 2.5 | 1961.4 | 1961.0 | 1961.8 | 0.8 |
| MEUSE | 48 | 47 | 49 | 2.6 | 14.4 | 63.5 | 22.2 | 0.58 | 46.0 | 45.4 | 2.5 | 1960.6 | 1960.2 | 1961.0 | 0.8 |
| MEUSE | 50 | 49 | 51 | 2.7 | 16.5 | 78.4 | 14.2 | 0.7 | 48.0 | 41.3 | 2.5 | 1959.8 | 1959.4 | 1960.2 | 0.8 |
| MEUSE | 52 | 51 | 53 | 2.8 | 16.6 | 76.8 | 8.1 | 0.49 | 46.0 | 40.8 | 2.5 | 1959.0 | 1958.6 | 1959.4 | 0.8 |
| MEUSE | 54 | 53 | 55 | 2.8 | 15.9 | 73.4 | 3.57 | NA | 45.0 | 41.6 | 2.5 | 1958.2 | 1957.8 | 1958.6 | 0.8 |
| MEUSE | 56 | 55 | 57 | 3.0 | 18.9 | 99.2 | 1.85 | NA | 40.6 | 39.6 | 2.5 | 1957.4 | 1957.0 | 1957.8 | 0.8 |
| MEUSE | 58 | 57 | 59 | 3.2 | 19.9 | 119.2 | 0.95 | NA | 38.1 | 36.8 | 2.5 | 1956.6 | 1956.2 | 1957.0 | 0.8 |
| MEUSE | 60 | 59 | 61 | 3.1 | 19.5 | 97.0 | 0.57 | NA | 39.0 | 34.5 | 2.5 | 1955.8 | 1955.4 | 1956.2 | 0.8 |
| MEUSE | 62 | 61 | 63 | 3.1 | 18.4 | 81.5 | 0.34 | NA | 41.7 | 36.7 | 2.5 | 1955.0 | 1954.6 | 1955.4 | 0.8 |
| MEUSE | 64 | 63 | 65 | 3.1 | 18.0 | 91.5 | <LD | NA | 43.5 | 38.8 | 2.5 | 1954.2 | 1953.8 | 1954.6 | 0.8 |
| MEUSE | 66 | 65 | 67 | 2.7 | 16.5 | 90.1 | 0.31 | NA | 41.2 | 39.8 | 2.5 | 1953.4 | 1953.0 | 1953.8 | 0.8 |
| MEUSE | 68 | 67 | 69 | 2.7 | 18.2 | 114.0 | 0.33 | NA | 40.6 | 36.6 | 2.5 | 1952.6 | 1952.2 | 1953.0 | 0.8 |
| MEUSE | 70 | 69 | 71 | 3.1 | 19.8 | 126.9 | 0.32 | NA | 40.4 | 37.5 | 2.5 | 1951.8 | 1951.4 | 1952.2 | 0.8 |
| MEUSE | 72 | 71 | 73 | 2.8 | 19.6 | 151.0 | <LD | NA | 39.4 | 34.7 | 2.5 | 1951.0 | 1950.6 | 1951.4 | 0.8 |
| MEUSE | 74 | 73 | 75 | 3.4 | 30.7 | 211.8 | 0.23 | NA | 39.0 | 30.9 | 2.5 | 1950.2 | 1949.8 | 1950.6 | 0.8 |
| MEUSE | 76 | 75 | 77 | 3.0 | 20.0 | 135.0 | <LD | NA | 36.7 | 32.7 | 2.5 | 1949.4 | 1949.0 | 1949.8 | 0.8 |
| MEUSE | 78 | 77 | 79 | 2.9 | 19.4 | 142.2 | <LD | NA | 42.2 | 34.5 | 2.5 | 1948.6 | 1948.1 | 1949.1 | 1.0 |
| MEUSE | 80.75 | 79.875 | 79 | 3.5 | 24.4 | 159.9 | <LD | NA | 37.1 | 31.0 | 2.5 | 1947.5 | 1947.0 | 1948.1 | 1.1 |

| **River** | **Mean Depth (cm)** | **Min Depth (cm)** | **Max Depth (cm)** | **D10 (µm)** | **D50 (µm)** | **D90 (µm)** | **^137^Cs (Bq/kg)** | **^241^Am (Bq/kg)** | **^210^Pb (Bq/kg)** | **^214^Bi (Bq/kg)** | **ASR (cm/y)** | **Mean age (y)** | **Min age (y)** | **Max age (y)** | **IP (y)** |
| --- | --- | --- | --- | --- | --- | --- | --- | --- | --- | --- | --- | --- | --- | --- | --- |
| MOSELLE | 4 | 0 | 8 | 3.4 | 25.9 | 122 | 8.3 | 0.068 | 118 | 48.1 | 0.7 | 2016.0 | 2011.8 | 2020.2 | 8.4 |
| MOSELLE | 11.75 | 8 | 15.5 | 4.4 | 36.4 | 270.2 | 7.5 | NA | 125 | 47.6 | 0.7 | 2004.9 | 1999.7 | 2010.2 | 10.5 |
| MOSELLE | 18.75 | 15.5 | 22 | 4.0 | 34.5 | 366.7 | 7.7 | NA | 104 | 46.3 | 0.7 | 1994.9 | 1990.2 | 1999.7 | 9.5 |
| MOSELLE | 25 | 22 | 28 | 3.9 | 37.0 | 844.8 | 11.6 | 0.0553 | 73 | 47.4 | 0.7 | 1986.0 | 1982.6 | 1989.4 | 6.8 |
| MOSELLE | 30 | 28 | 32 | 4.6 | 43.9 | 163.8 | 2.59 | NA | 52 | 40.5 | 1.08 | 1981.4 | 1979.3 | 1983.4 | 4.1 |
| MOSELLE | 33.75 | 32 | 35.5 | 3.5 | 25.4 | 85.8 | 3.65 | 0.073 | 89 | 47.6 | 1.08 | 1977.9 | 1975.8 | 1979.9 | 4.1 |
| MOSELLE | 38.75 | 35.5 | 42 | 3.4 | 24.1 | 82.6 | 8.7 | 0.229 | 69 | 48.4 | 1.08 | 1973.2 | 1970.7 | 1975.7 | 5.0 |
| MOSELLE | 44.5 | 42 | 47 | 2.9 | 19.0 | 62.0 | 18.1 | 0.66 | 73 | 47 | 1.08 | 1967.9 | 1965.3 | 1970.4 | 5.1 |
| MOSELLE | 49.75 | 47 | 52.5 | 2.6 | 16.9 | 57.0 | 25.7 | 0.97 | 74 | 47 | 1.08 | 1963.0 | 1961.6 | 1964.4 | 2.8 |
| MOSELLE | 56.25 | 52.5 | 60 | 2.6 | 16.8 | 52.5 | 10.9 | 0.236 | 63 | 47.1 | 7.84 | 1962.2 | 1961.8 | 1962.6 | 0.8 |
| MOSELLE | 62.5 | 60 | 65 | 3.2 | 21.7 | 93.3 | 7.6 | 0.209 | 69 | 46.4 | 7.84 | 1961.4 | 1961.0 | 1961.7 | 0.7 |
| MOSELLE | 67.5 | 65 | 70 | 2.9 | 18.5 | 62.8 | 15.8 | 0.447 | 63 | 47.5 | 7.84 | 1960.7 | 1960.4 | 1961.1 | 0.7 |
| MOSELLE | 72.5 | 70 | 75 | 2.9 | 16.9 | 48.6 | 7.5 | 0.5 | 74 | 45.4 | 7.84 | 1960.1 | 1959.8 | 1960.4 | 0.6 |
| MOSELLE | 77.5 | 75 | 80 | 2.9 | 17.9 | 53.6 | 6.5 | 0.33 | 71 | 47.8 | 7.84 | 1959.5 | 1959.1 | 1959.8 | 0.7 |
| MOSELLE | 82.5 | 80 | 85 | 3.0 | 17.9 | 54.6 | 8.4 | NA | 75 | 47.1 | 7.84 | 1958.8 | 1958.5 | 1959.1 | 0.6 |
| MOSELLE | 87.5 | 85 | 90 | 3.1 | 18.9 | 57.1 | 10.5 | NA | 72 | 42.6 | 7.84 | 1958.2 | 1957.9 | 1958.5 | 0.6 |
| MOSELLE | 92.5 | 90 | 95 | 3.7 | 22.8 | 65.7 | 11.4 | NA | 72 | 47.7 | 7.84 | 1957.5 | 1957.2 | 1957.9 | 0.7 |
| MOSELLE | 97.5 | 95 | 100 | 3.3 | 23.1 | 71.9 | 9.2 | 0.59 | 79 | 44 | 7.84 | 1956.9 | 1956.6 | 1957.2 | 0.6 |
| MOSELLE | 102.5 | 100 | 105 | 2.7 | 18.7 | 59.1 | 9.5 | 0.61 | 82 | 45 | 7.84 | 1956.3 | 1956.0 | 1956.6 | 0.6 |
| MOSELLE | 107.5 | 105 | 110 | 3.2 | 19.6 | 55.7 | 4.7 | NA | 81 | 39.1 | 7.84 | 1955.6 | 1955.3 | 1956 | 0.7 |
| MOSELLE | 112.5 | 110 | 115 | 2.7 | 19.4 | 67.4 | 1.2 | NA | 63 | 50.9 | 7.84 | 1955.0 | 1954.1 | 1955.8 | 1.7 |
| MOSELLE | 117.5 | 115 | 120 | 2.7 | 19.4 | 67.6 | <LD | 0.0021 | 64 | 50.8 | 1.81 | 1952.2 | 1950.9 | 1953.5 | 2.6 |
| MOSELLE | 122 | 120 | 124 | 2.7 | 20.6 | 87.8 | <LD | NA | 58 | 50.1 | 1.81 | 1949.7 | 1948.4 | 1951.0 | 2.6 |
| MOSELLE | 127 | 124 | 130 | 3.4 | 37.8 | 203.0 | <LD | NA | 47 | 42.3 | 1.81 | 1947.0 | 1945.2 | 1948.7 | 3.5 |
| MOSELLE | 134.5 | 130 | 139 | 6.9 | 141.8 | 296.7 | <LD | NA | 36 | 31.4 | 1.81 | 1942.8 | 1940.8 | 1944.9 | 4.1 |
| MOSELLE | 142 | 139 | 145 | 73.6 | 187.7 | 371.2 | <LD | NA | 34.2 | 31.7 | 1.81 | 1938.7 | 1936.7 | 1940.7 | 4.0 |
| MOSELLE | 149.25 | 145 | 153.5 | 5.6 | 120.8 | 302.8 | <LD | NA | 39 | 32.3 | 1.81 | 1934.7 | 1932.7 | 1936.7 | 4.0 |
| MOSELLE | 156.75 | 153.5 | 160 | 6.2 | 121.1 | 338.1 | <LD | NA | 37 | 34.6 | 1.81 | 1930.6 | 1928.6 | 1932.5 | 3.9 |
| MOSELLE | 163.5 | 160 | 167 | 25.7 | 207.8 | 541.9 | <LD | NA | 42 | 37.1 | 1.81 | 1926.8 | 1925 | 1928.7 | 3.7 |

**References for SI:**

1. Oudin, L.O., Reyes-Marchant, P., Vigneron, T., J.E., R., Lair, N., J.F, M., J.P., B., Descy, J.-P., Leitão, M., P., S. & Bacchi, M. The Loire basin. pp. 167-181 (2009).
2. Dendievel, A.-M., Grosbois, C., Ayrault, S., Evrard, O., Coynel, A., Debret, M., Gardes, T., Euzen, C., Schmitt, L., Chabaux, F., Winiarski, T., Van Der Perk, M. & Mourier, B. Key factors influencing metal concentrations in sediments along Western European Rivers: A long-term monitoring study (1945–2020). *Science of The Total Environment* **805**, 149778 (2022). <https://doi.org/10.1016/j.scitotenv.2021.149778>

(3) Belleudy, P. Restoring flow capacity in the Loire River bed. *Hydrological Processes*, **14**, 2331–2344 (2000). [https://doi.org/10.1002/1099-1085(200009)14:13<2331::AID-HYP32>3.0.CO;2-6](https://doi.org/10.1002/1099-1085(200009)14:13%3c2331::AID-HYP32%3e3.0.CO;2-6).

(4) Rodrigues, S., Bréhéret, J.-G., Macaire, J.-J., Moatar, F., Nistoran, D. & Jugé, P. Flow and sediment dynamics in the vegetated secondary channels of an anabranching river: The Loire River (France). *Sedimentary Geology* **186**, 89–109 (2006). <https://doi.org/10.1016/j.sedgeo.2005.11.011>

(5) Olivier, J.-M., Carrel, G., Lamouroux, N., Dole-Olivier, M.-J., Malard, F., Bravard, J.-P., Piégay, H., Castella, E. & Barthélemy, C. Chapter 7 - The Rhône River Basin, in: Tockner, K., Zarfl, C., Robinson, C.T. (Eds.), Rivers of Europe (Second Edition). Elsevier, pp. 391–451 (2009). <https://doi.org/10.1016/B978-0-08-102612-0.00007-9>

(6) Tena, A., Piégay, H., Seignemartin, G., Barra, A., Berger, J.F., Mourier, B. & Winiarski, T. Cumulative effects of channel correction and regulation on floodplain terrestrialisation patterns and connectivity. *Geomorphology* **354**, 107034 (2020). <https://doi.org/10.1016/j.geomorph.2020.107034>

(7) Riquier, J. Réponses hydrosédimentaires de chenaux latéraux restaurés du Rhône français. Université de Lyon, France (2015).

(8) Bravard, J.-P. Discontinuities in braided patterns: The River Rhône from Geneva to the Camargue delta before river training. *Geomorphology* **117**, 219–233 (2010).

(9) Seignemartin, G., Mourier, B., Riquier, J., Winiarski & T. Piégay, H., Dike fields as drivers and witnesses of twentieth century hydrosedimentary changes in a highly engineered river (Rhône River, France). *Geomorphology,* **431**, 108689 (2023). <https://doi.org/10.1016/j.geomorph.2023.108689>

(10) Schmitt, L., Beisel, J.-N., Preusser, F., de Jong, C., Wantzen, K. M., Chardon, V., … Brackhane, S. Sustainable Management of the Upper Rhine River and Its Alluvial Plain: Lessons from Interdisciplinary Research in France and Germany, In : Hamman Ph., Vuilleumier S., Sustainability Research in the Upper Rhine Region : Concepts and case studies, Etudes Alsaciennes et Rhénanes, Presses Universitaires de Strasbourg, p. 201-226 (2019).

(11) Schmitt, L. Typologie hydro-géomorphologique fonctionnelle de cours d’eau : Recherche méthodologique appliquée aux systèmes fluviaux d’Alsace (Phdthesis). Université Louis Pasteur, Strasbourg (2001).

(12) Euzen C., Chabaux F., Rixhon G., Preusser F., Eyrolle F., Chardon V., Zander A. M., Badariotti D., Schmitt L. Innovative multi-method geochronological approach to decipher (sub-)modern floodplain sedimentation (Upper Rhine, France). *Quaternary Geochronology*, 101561 (2024) <https://doi.org/10.1016/j.quageo.2024.101561>

(13) Chardon, V., Herrault, P.-A., Staentzel, C., Skupinski, G., Finance, O., Wantzen, K.-M., Schmitt, L. Using transition matrices to assess the spatio-temporal land cover and ecotone changes in fluvial landscapes from historical planimetric data. *Earth Surface Processes and Landforms*, **47** (11) 2647-2659 (2022) <https://doi-org.scd-rproxy.u-strasbg.fr/10.1002/esp.5437>

(14) Flipo, N., Lestel, L., Labadie, P., Meybeck, M. & Garnier, J. Trajectories of the Seine River Basin. Springer International Publishing, p. 1 (2020). <https://doi.org/10.1007/698_2019_437>

(15) Descy, J.-P., Kestemont, P., Everbecq, E., Verniers, G., Usseglio-Polatera, P., Gérard, P., Viroux, L., Beisel, J.-N. & Smitz, J.-S. The Meuse River Basin. Rivers of Europe 154–165 (2009).

(16) Grevilliot, F., Broyer, J. & Muller, S., Phytogeographical and phenological comparison of the Meuse and the Saône valley meadows (France). *Journal of Biogeography*, **25**, 339–360 (1998). <https://doi.org/10.1046/j.1365-2699.1998.252165.x>

(17) Wantzen, K.M., Uehlinger, U., Van der Velde, G., Leuven, R.S.E.W., Schmitt, L. & Beisel, J.-N., Chapter 10 - The Rhine River basin, in: Tockner, K., Zarfl, C., Robinson, C.T. (Eds.), Rivers of Europe (Second Edition). Elsevier, pp. 333–391 (2022). <https://doi.org/10.1016/B978-0-08-102612-0.00010-9>

(18) Le Meur, M.L. Matières en suspension de la Moselle (Lorraine, France) : caractérisation minérale et organique et réactivité vis-à-vis des contaminants métalliques (PhD thesis). Université de Lorraine (2016).

(19) Berger, F. Les enjeux de la canalisation de la Moselle et de la Sarre jusqu’au Rhin pour les industriels sidérurgistes du bassin Lorraine-Sarre-Luxembourg (jusqu’aux années 1950). Archives nationales du monde du travail, p. 137 (2015).
